# Structural changes in alginate-based microspheres exposed to *in vivo* environment as revealed by confocal Raman microscopy

**DOI:** 10.1038/s41598-018-20022-y

**Published:** 2018-01-26

**Authors:** Zuzana Kroneková, Michal Pelach, Petra Mazancová, Lucia Uhelská, Dušana Treľová, Filip Rázga, Veronika Némethová, Szabolcs Szalai, Dušan Chorvát, James J. McGarrigle, Mustafa Omami, Douglas Isa, Sofia Ghani, Eva Majková, José Oberholzer, Vladimír Raus, Peter Šiffalovič, Igor Lacík

**Affiliations:** 1 Department for Biomaterials Research, Polymer Institute of the Slovak Academy of Sciences, Dúbravská cesta 9, 845 41 Bratislava, Slovakia; 2 Department of Multilayers and Nanostructures, Institute of Physics of the Slovak Academy of Sciences, Dúbravská cesta 9, 845 11 Bratislava, Slovakia; 3 Institute of Macromolecular Chemistry, Academy of Sciences of the Czech Republic, Heyrovsky Sq. 2, 162 06 Prague 6, Czech Republic; 4 Department of Biophotonics, International Laser Center, Ilkovicova 3, 841 04 Bratislava, Slovakia; 5 Division of Transplantation, Department of Surgery, University of Illinois at Chicago, 840 South Wood Street, Chicago, Illinois 60612 USA

## Abstract

A next-generation cure for type 1 diabetes relies on immunoprotection of insulin-producing cells, which can be achieved by their encapsulation in microspheres made of non-covalently crosslinked hydrogels. Treatment success is directly related to the microsphere structure that is characterized by the localization of the polymers constituting the hydrogel material. However, due to the lack of a suitable analytical method, it is presently unknown how the microsphere structure changes *in vivo*, which complicates evaluation of different encapsulation approaches. Here, confocal Raman microscopy (CRM) imaging was tailored to serve as a powerful new tool for tracking structural changes in two major encapsulation designs, alginate-based microbeads and multi-component microcapsules. CRM analyses before implantation and after explantation from a mouse model revealed complete loss of the original heterogeneous structure in the alginate microbeads, making the intentionally high initial heterogeneity a questionable design choice. On the other hand, the structural heterogeneity was conserved in the microcapsules, which indicates that this design will better retain its immunoprotective properties *in vivo*. In another application, CRM was used for quantitative mapping of the alginate concentration throughout the microbead volume. Such data provide invaluable information about the microenvironment cells would encounter upon their encapsulation in alginate microbeads.

## Introduction

With ever increasing numbers of diabetic patients worldwide, there is a strong focus towards the identification of a functional treatment for diabetes that would complement or replace the current treatment of exogenous insulin administration. Continuous glucose control by immunoprotected insulin-producing cells is seen as the next-generation treatment for the type 1 diabetes^1–3^, with several clinical trials having already been performed. The immunoprotection of cells is provided by hydrogel-based semipermeable membranes that can take various forms such as conformal coating^1^, microspheres^4–7^, and macrocapsules^8^. Of the hydrogel materials available, non-covalently crosslinked, alginate-based hydrogels are the most widely employed because their application offers experimental simplicity, design flexibility, and maintenance of favorable physiological conditions for the cells during the encapsulation process. Nevertheless, a number of questions remain unanswered relating to the interplay between the properties of the hydrogel-made semipermeable membrane and the overall response of the immune system to the implant, ultimately determining the functionality of transplanted cells^1,2,9–11^. The properties of the semipermeable membrane are given not only by its chemical composition but also by the hydrogel structure, i.e., the spatial localization of its constituents. Unfortunately, the field currently lacks a straightforward, non-invasive method for evaluating structural changes of the hydrogel material in response to the surrounding environment. As a result, cell performance is typically correlated only with hydrogel characteristics determined prior to implantation, which disregards the possibility that the *in vivo* environment may substantially alter the functional characteristics of the implant. Such an approach can lead to misleading conclusions. A rational design of the hydrogel properties is thus problematic, which creates obstacles to the advancement of the encapsulation concept to clinical trials.

The key structural characteristic of the non-covalently crosslinked hydrogel material constituting the microsphere and providing immunoprotection to cells is the spatial distribution of the polymeric components, i.e., the local concentrations of polymeric chains within the 3D space of a microsphere. The spatial distribution is thought to have a direct impact on the mechanical and chemical stability of the microsphere, diffusion properties of the hydrogel, and also the local microenvironment of encapsulated cells^12–17^. These factors are closely related to immunoprotection of transplanted cells as well as to implant biotolerance and function. During the last two decades, a number of physico-chemical methods aimed at characterization of hydrogel microspheres have emerged^18^. However, still there is no practical method for the characterization of polymer spatial distribution in microspheres under physiological conditions that is sufficiently sensitive, non-invasive, label-free, applicable to explanted microspheres, and does not require sample pre-treatment. Confocal fluorescence laser scanning microscopy (CLSM) is the current state-of-the-art method, fulfilling some of these criteria^19^. It was used for a number of purposes^18^, including visualization of spatial distribution of polymers and gelling ions in alginate-based microspheres^20^. In CLSM, a tightly focused laser beam is scanned across the analyzed specimen, and the localized fluorescence signal is continuously detected through a confocal pinhole placed in the image plane. The principal disadvantage of CLSM is the necessity to fluorescently label the sample. Sample labeling can be relatively straightforward for simple single-component systems, such as alginate microbeads. However, for multi-component microsphere designs, e.g., microcapsules, this approach becomes laborious, and parallel detection of individual polymers might not be feasible. In addition, when the labeled hydrogel material is implanted into an *in vivo* environment, the fluorescent label may inflict unwanted interactions and alter thus the implant performance. Again, this risk is higher for fully labeled multi-component microsphere designs. Perhaps due to these drawbacks, CLSM has not been considered for study of the structure of implanted microspheres^18^. It is thus currently unknown if and how the microsphere structure changes *in vivo* and other environments.

We postulate here that the limitations associated with CLSM analysis of microspheres can be conveniently overcome by the use of confocal Raman microscopy (CRM). The working principle of CRM is derived from that of CLSM; however, chemical composition of the sample is obtained from the Raman spectrum measured in a probed confocal volume, and thus no labeling is necessary. Even though the Raman signal in CRM is significantly weaker than the fluorescence signal in CLSM, this shortcoming can be largely compensated by the latest technological developments in CRM detection schemes^21,22^. Indeed, the methodological advancements have recently brought about a rapid expansion of CRM applications in various fields such as pharmacology, microbiology, toxicology, or human biology^22^. Therefore, it comes as a surprise that CRM has not been exploited for the characterization of hydrogel materials used for immunoprotection of transplanted cells. In a rare recent example, Vegas *et al*. utilized CRM to localize chemically modified alginate in dried alginate microbeads^23^. However, the study neglected the main advantage of CRM, i.e., the analysis under physiological conditions. Furthermore, Heinemann *et al*. used a similar, Raman spectroscopy-based method for qualitative and quantitative characterization of alginate spatial distribution in large alginate beads in the aqueous environment^24^. This work was largely methodological, though, envisioning applications in the study of hydrogel network formation and diffusion of small molecules in alginate matrices. The huge potential of CRM in the encapsulation field has thus remained untapped.

This work represents the first in depth study describing application of CRM imaging to characterization of microspheres aimed at cell encapsulation. Here, empty microspheres were studied to assess the influence of the environment on the microsphere structure (i.e., on the hydrogel material), removing the potential influence of the cell presence (e.g., metabolic activity effects). In order to establish CRM as a reliable method of microsphere characterization, it was validated through side-by-side comparison with CLSM, various technical issues were addressed, and usability of the quantitative mode was verified for the target hydrogel materials. Subsequently, to illustrate the great utility of CRM imaging on real-world examples, it was applied to two major microsphere designs used in the cell encapsulation field: the single-component ionotropically gelled alginate microbeads^5,6,23,25^ and the three-component polyelectrolyte complex-based microcapsules^26^. This allowed us to quantitatively map the true alginate concentrations throughout the volume of alginate microbeads of different heterogeneity. Furthermore, CRM imaging was employed to determine how the microsphere structure changes upon microsphere implantation. This made possible the first-of-its-kind, side-by-side comparison of microbead and microcapsule designs. Remarkable differences between these two encapsulation systems were revealed, and the possible consequences for their immunoprotective properties are discussed here. Note that in this work the term microsphere is used as a universal term for systems used for microencapsulation of cells, the term microcapsule for an encapsulation system consisting of two or more polymers forming the membrane, and the term microbead for the encapsulation system typically made of one polymer forming a continuous hydrogel of varying degree of heterogeneity^18^.

## Experimental Section

### Materials

Ultrapure high G content and low viscosity sodium alginate (UP-LVG) of 58 mol.% guluronic acid content (determined by ^1^H NMR) was purchased from Novamatrix (lot # FP-606-02, Sandvika, Norway). High viscosity sodium alginate (SA) of 39 mol.% guluronic acid content (determined by ^1^H NMR) and of *M*_w_ = 235 kDa, *M*_n_ = 118 kDa (determined by SEC with MALLS detection) was supplied by ISP Alginates (Girvan, Ayrshire, UK) and purified following the procedure described by de Vos *et al*.^27^. The residual water content for UP-LVG and SA determined by gravimetry was 17 and 10 wt.%, respectively. Sodium cellulose sulfate (SCS) of the degree of substitution of 2.4 and *M*_w_ = 760 kDa (determined by viscometry^28^) was from Acros Organics (NJ, USA). Poly(methylene-co-cyanoguanidine) hydrochloride (PMCG) was supplied by Scientific Polymer Products, Inc. (Ontario, NY, USA) as a ca 35% aqueous solution and was freeze-dried before use. NaCl, BaCl_2_.2H_2_O, CaCl_2_.2H_2_O, D-mannitol, phosphate buffer saline (PBS), 3-(*N*-morpholino)propanesulfonic acid buffer (MOPS), 1-ethyl-3-(3-dimethyl aminopropyl) carbodiimide hydrochloride (EDC), fluorescein amine, rhodamine B isothiocyanate (RBITC), and *N*-hydroxysulfosuccinimide sodium salt (sulfo-NHS) were used as received. The ultrapure water (18 MΩ.cm) was used for preparation of solutions.

## Methods

### CRM Instrumentation

All experiments were performed on the WITec alpha 300 R+ confocal microscope, equipped with the WITec UHTS300 spectrometers, at a controlled room temperature of 23 °C. The water immersion objective Carl Zeiss 20×/1NA was used for the analysis of alginate microbeads, multi-component microcapsules and planar alginate hydrogels. The 785 nm and 532 nm laser lines were used for excitation of Raman signal. The parameters for the 785 nm laser line were: the power at the sample 100 mW, the average flux density in probed confocal volume 1.63 mW/μm^3^, lateral resolution 0.98 ± 0.04 μm, axial resolution 20.2 ± 0.1 μm. The parameters for the 532 nm laser line were: the power at the sample 32 mW, the average flux density in the probed confocal volume 0.91 mW/μm^3^, lateral resolution 0.86 ± 0.08 μm, axial resolution 15.2 ± 0.9 μm. Both the excitation laser lines were evaluated with regard to effects of autofluorescence emission and laser parameters (Supplementary Fig. S1). The optical grating with grooves density of 600 lines/mm was used to disperse the Raman spectra. The spectra collection time was typically in the range of 3 to 10 s using a line scan with the step-wise scanning of a step size from 1 to 8 µm, depending on the required level of resolution. The overall time to create the CRM intensity profile of the respective polymer along the equatorial microsphere cross-section varied between 40 and 60 min. The Carl Zeiss 10×/1NA objective was used to analyze polymers in a powder form. Before the measurements were carried out, the system energy check was performed using isopropyl alcohol. The laser line 532 nm was also employed to acquire fluorescent signal of fluorescein amine- and rhodamine B isothiocyanate-labeled polymers with the power reduced to about 100 μW. The instrument was controlled by WITec Suite software. The workflow diagram from data acquisition to evaluation is shown in Supplementary Fig. S2 and the details on data processing are described in Supplementary Note 1. A custom-designed holder for hydrogel microspheres made of silicone was employed in order to fix the position of microspheres during the CRM analysis (Supplementary Fig. S3). A plastic Pasteur pipette with the diameter of 2 mm was used to position individual microspheres to the wells of the holder. The objective was focused at the equatorial microsphere cross-section and the line scan was performed from left to right (Supplementary Fig. S3).

### CRM analysis of microsphere components

In order to discriminate the molecular signals from the spectral noise and to define proper conditions for Raman spectra acquisition, the analysis of a polymer in a powder form was performed first. These spectra were used as a reference for determining the peak positions in solution where the signal may be significantly weakened. The comparison between Raman intensity spectra for polymers in a powder form and in solution, respectively, is shown in Supplementary Fig. S4 for sodium alginate and in Supplementary Fig. S5 for SCS and PMCG. Supplementary Fig. S6 shows the Raman spectra of the SA-SCS/PMCG microcapsule and highlights the bands we used for following the spatial polymer distribution in this work (1415 rel. cm^−1^ for SA, 1070 rel. cm^−1^ for SCS, and 770 rel. cm^−1^ for PMCG).

### CRM analysis of microbeads and microcapsules

The data obtained from the CRM imaging of microspheres are presented as relative (alginate microbeads and multi-component microcapsules) and absolute (alginate microbeads) concentration profiles, where in the latter case a calibration curve was applied. The conditions for obtaining the calibration curve (Supplementary Fig. S7) are described in Supplementary Note 2. In case of imaging of the planar alginate hydrogels (Supplementary Fig. S8), each slab was measured twice by scanning the areas of 1 × 1 mm^2^ and 0.1 × 0.1 mm^2^, respectively. In all Raman measurements, the Raman signal intensity from alginate was normalized to the signal intensity of water to suppress absorption effects and focal volume fluctuations (Supplementary Fig. S9). When the microbeads were imaged in D-mannitol solution, the Raman spectrum of D-mannitol was subtracted from the overall Raman spectra (Supplementary Fig. S10). At least 6 different microspheres were analyzed for each microsphere type.

### CLSM Instrumentation

Confocal laser scanning microscope (CLSM) LSM510 META on Axiovert 200 (both Zeiss) using 10×/0.45NA Apochromat objective was used to monitor the spatial distribution of fluorescently labeled polymers within the microspheres. Optical setup to determine fluorescence of the fluorescein amine-labeled SA: 488 nm laser line (100% relative power), 488 nm main dichroic mirror, emission filter long-pass 505 nm. Optical setup to determine fluorescence of the rhodamine B-labeled PMCG: 543 nm laser line (100% relative power), 488 nm main dichroic mirror, emission filter long-pass 560 nm.

### Covalent labeling of SA and PMCG

SA was fluorescently labeled by fluorescein amine following the protocol described by Strand *et al*.^20^. PMCG was fluorescently labeled by RBITC. RBITC at the concentration of 0.0011 mM was stirred in 50 mL of 10 wt.% aqueous PMCG solution for 18 hours at pH 7 and room temperature. Labeled PMCG was isolated by repeated precipitation to absolute ethanol and dried in vacuum oven at 40 °C. The fluorescently labeled SA and PMCG were kept at −4 °C and protected from light until use.

### Preparation of alginate microbeads using fluorescently labeled SA

**Figure 1:**
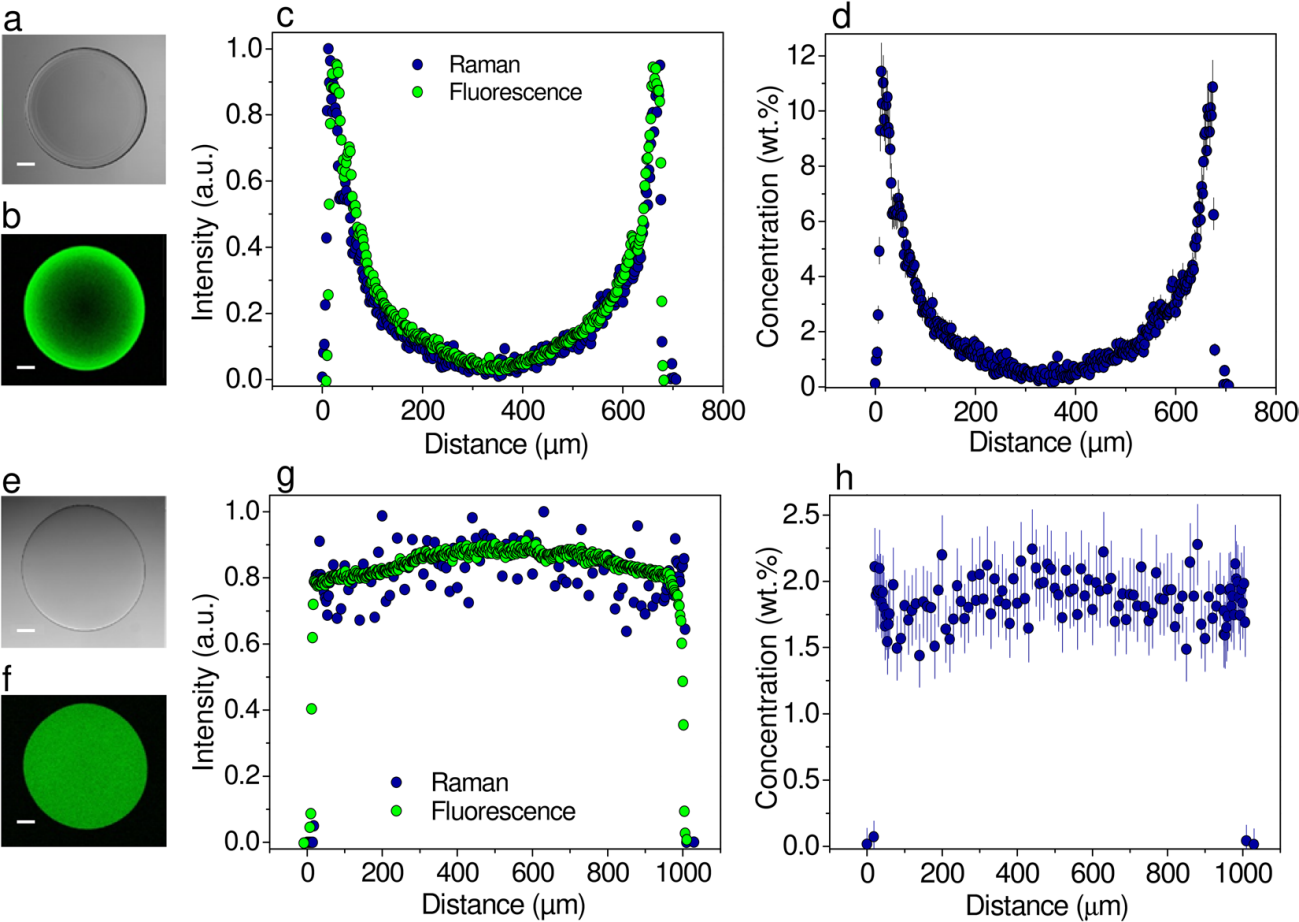
CRM and CLSM imaging of alginate microbeads with heterogeneous (**a**–**d**) and homogeneous (**e**–**h**) spatial distribution of alginate. (**a**,**e**) CLSM image in the transmission mode. (**b**,**f**) CLSM image in the fluorescence emission mode at the equatorial microbead cross-section. (**c**,**g**) Overlay of CLSM and CRM intensity profiles at the equatorial microbead cross-section. (**d**,**h**) Spatial distribution of alginate in the microbeads shown in (**c**) and (**g**), respectively, expressed as the absolute alginate concentration in wt.%. Bars in (**a**,**b**,**e**,**f**) are equal to 100 μm.

SA microbeads with homogeneous and heterogeneous spatial distribution of alginate (data shown in Fig. 1) were prepared following the literature protocols^5,15,20,29,30^. The custom designed coaxial nozzle, which operates *via* air-stripping, was used to generate alginate microbeads in the size range of 600 to 800 μm. Alginate microbeads with the homogeneous spatial distribution of alginate were made of 1.8 wt.% fluorescently labeled SA dissolved in saline. Droplets of alginate solution were controllably dropped into 100 mL of saline containing CaCl_2_ (100 mM). The collection and gelling times were 1 min and 7 min, respectively. The microbeads were then washed with saline three times and stored in 2 mM CaCl_2_ solution in saline in a refrigerator. Alginate microbeads with the heterogeneous spatial distribution of alginate were made of 1.8 wt.% fluorescently labeled SA dissolved in 0.3 M solution of D-mannitol. The gelling solution was 10 mM BaCl_2_ in 0.15 M solution of D-mannitol. The collection and gelling times were 1 min and 7 min, respectively. Microbeads were washed with 0.15 M solution of D-mannitol three times and stored in refrigerator in 2 mM CaCl_2_ in 0.15 M D-mannitol. The pH of all solutions was adjusted to 7.4.

### Preparation of sterile alginate microbeads for implantation studies and determination of the effect of environment on spatial distribution of alginate

**Figure 2:**
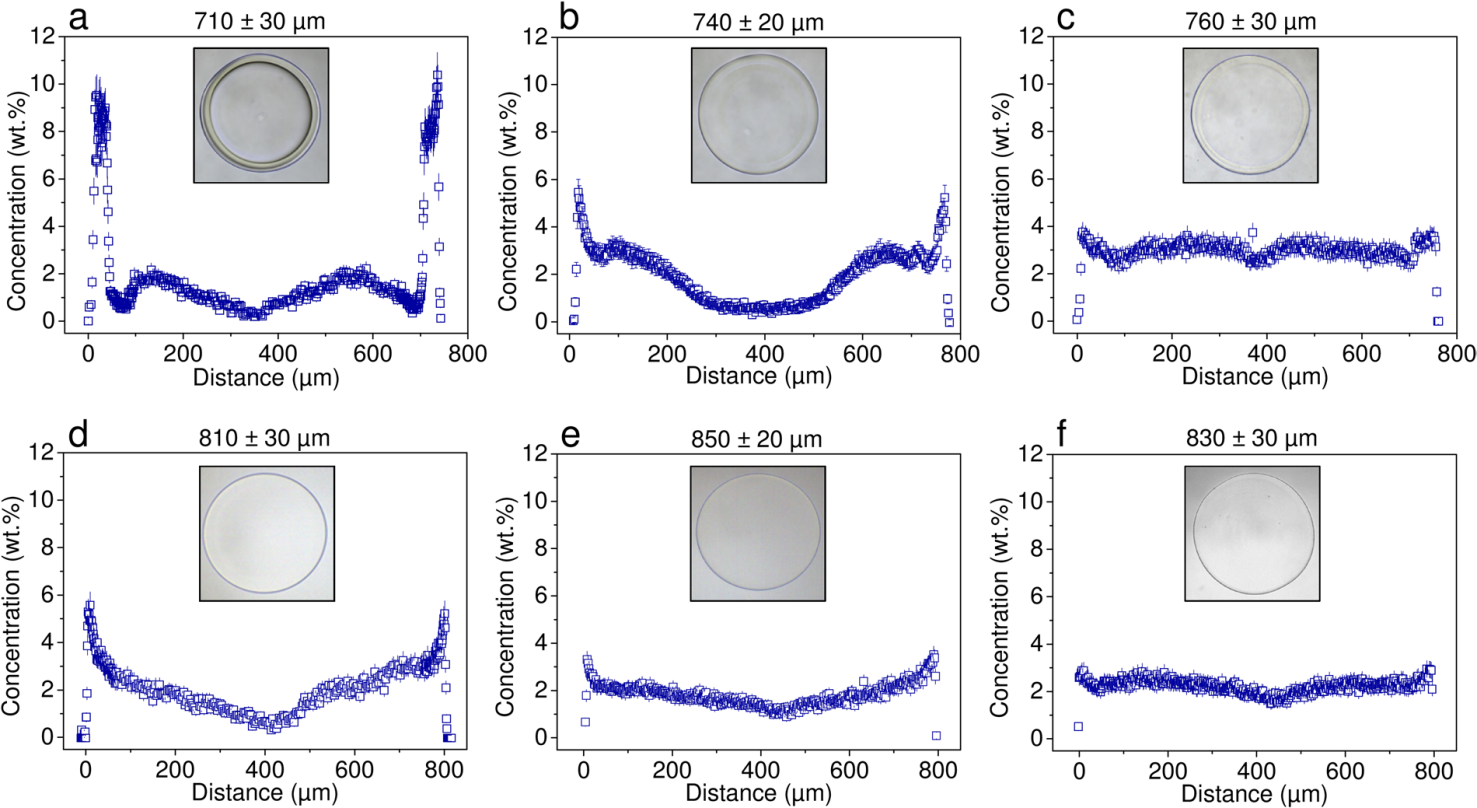
The effect of environment on spatial distribution of alginate (in wt.%) in alginate microbeads of higher (**a**–**c**) and lower (**d**–**f**) initial heterogeneity as revealed by quantitative CRM imaging. (**a**,**d**) Microbeads after preparation (in D-mannitol). (**b**,**e**) Microbeads stored for 24 hours in saline at 37 °C. (**c**,**f**) Microbeads after explantation from nude mice 4 weeks post-intraperitoneal implantation (stored in saline with 2 mM CaCl_2_). The optical microscopy images are included to visualize the particular alginate microbeads characterized by CRM imaging. The numbers refer to the microbead diameter.

Alginate microbeads differing in spatial distribution of alginate (Fig. 2 and Supplementary Fig. S11–S13) were prepared from 1.8 wt.% UP-LVG dissolved in 0.3 M solution of D-mannitol at pH 7.4 by air-stripping 4 mL of the solution into 400 mL of a gelling solution. The gelling solution for microbeads of higher heterogeneity contained 20 mM BaCl_2_ and 0.14 M D-mannitol in MOPS buffer (10 mM). The gelling solution for microbeads of lower heterogeneity contained 1 mM BaCl_2_, 50 mM CaCl_2_ and 0.14 M D-mannitol in MOPS buffer (10 mM). The collection time in gelling solution was 7 min, and the gelling time was additional 7 min. The microbeads were washed three times in 150 mL of 0.15 M solution of D-mannitol at pH 7.4 and stored in 0.3 M solution of D-mannitol with 2 mM CaCl_2_ at pH 7.4 and 4 °C. A small fraction of microbeads was subjected to CRM and the rest was transferred to a solution consisting of 2 mM CaCl_2_ in saline at pH 7.4. A small fraction of these microbeads was incubated at 37 °C for 24 h in this environment and then analyzed by CRM. The rest of microbeads (stored in saline with 2 mM CaCl_2_ at pH 7.4) were implanted intraperitoneally into nude mice. All solutions were 0.22 μm filter-sterilized using the syringe filters for alginate solutions and the bottle-top filters for gelling and washing solutions. Note that the used gelling protocols inherently lead to certain batch heterogeneity, which is illustrated in Supplementary Fig. S11; nevertheless, this does not influence the general trends in alginate spatial distribution observed by CRM (Supplementary Fig. S12 and S13).

### Preparation of planar alginate hydrogel slabs with homogeneous spatial distribution of alginate by the internal gelling method

Planar alginate hydrogel slabs were prepared from internally gelled SA through a protocol based on dissolution of CaCO_3_ as a source of calcium gelling ions in the acidic environment of slowly hydrolyzing d-(+)-glucono-δ-lactone (GDL)^31^. The targeted composition of the gelling system was 2 wt.% SA, 20 mM CaCO_3_ and 30 mM GDL in saline. 4.5 mL of 2.2 wt.% SA in saline was mixed with 10 mg of CaCO_3_ that was dispersed in 0.25 mL of saline by ultrasound PS04000 (Notus-Powersonics, Slovakia) for 3 min. Subsequently, 0.25 mL of 0.3 M GDL solution in saline was quickly added to the stirred solution. About 10 s after the GDL addition, the mixture was loaded between two glass plates of dimensions 20 × 50 × 1 mm, secured with an adhesive tape, and kept for 24 hours at 4 °C. Three hydrogel slabs were prepared in parallel from one gelling mixture. The slabs were immersed for 10 min to either saline or 5 mM CaCl_2_ or 50 mM CaCl_2_ and, subsequently, stored for at least 24 h in saline containing 5 mM CaCl_2_ prior to the CRM imaging. The resultant hydrogel slabs were weighed after each step to estimate the true alginate concentration in the hydrogel (changes due to swelling or shrinking in the storage solution). Details on these weights and resulting true alginate concentrations are contained in Supplementary Table S1.

### Preparation of sterile multi-component SA-SCS/PMCG microcapsules for implantation studies

**Figure 3:**
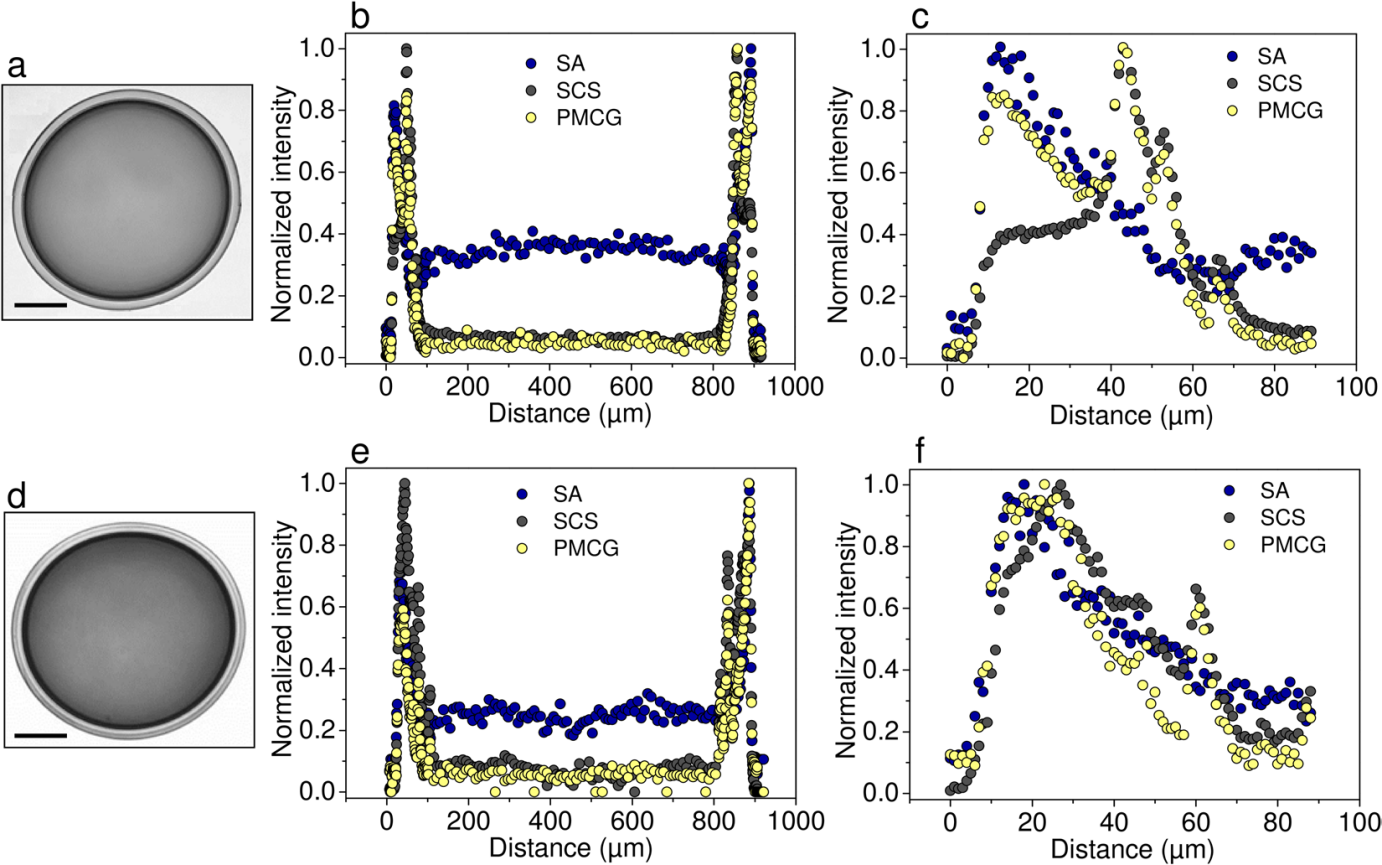
CRM intensity profiles (equatorial cross-section) of a multi-component microsphere (SA/SCS-PMCG) after preparation (**a**–**c**), and after explantation from the intraperitoneal space of C57bl/6 mice 2 weeks post-implantation (**d**–**f**). (**a**,**d**) Optical image (bar equals to 200 µm). Spatial distribution of individual polymeric components within the entire microcapsule (**b**,**e**) and in the outermost region only (**c**,**f**) obtained from Raman signal normalized to the maximum intensity.

Multi-component microcapsules (Fig. 3 and Supplementary Fig. S14) were prepared by employing the continuous 2-step protocol developed for preparation of SA-SCS/PMCG microcapsules^32^ with two multiloop reactors^33^ connected in a series. The SA-SCS solution (0.9 wt. % SA and 0.9 wt.% SCS dissolved in saline, pH 7.4) was air-stripped into droplets at the flow rate of 0.564 g/min using a custom designed coaxial nozzle. The droplets first fell into the funnel of the first reactor which was filled with a cation gelling solution of 1 mM BaCl_2_ and 50 mM CaCl_2_ dissolved in either 0.3 M D-mannitol (Fig. 3) or in saline (Supplementary Fig. S15), both at pH 7.4 (flow rate 23.3 g/min). The gelling time for each droplet in the first reactor was 20 s. Upon exiting the first reactor, the formed SA-SCS microbeads were carried by the gelling solution into the funnel of the second reactor that was continuously filled with a solution of 2.87 wt.% PMCG in saline (flow rate 22.8 g/min). The resulting PMCG concentration in the second reactor was 1.58 wt.%. The gelling time in the second reactor was 50 s. Microcapsules were collected in 200 mL of saline to stop the complexation reaction. This collecting solution was replaced by a fresh solution every 1 min. Pooled microcapsules after all collections were washed three times in saline and exposed to 50 mM citrate solution in saline at pH 7.4 for 10 min. Microcapsules were then washed three times in saline and stored at 4 °C. All the used solutions were 0.22 μm filter-sterilized using the syringe filter (SA-SCS solution) and the 0.22 μm bottle-top filters (gelling, washing and citrate solutions).

### Preparation of multi-component SA-CS/PMCG microcapsules with fluorescently labeled PMCG polycation

The preparation of sterile SA-CS/PMCG microcapsules was similar to that described for SA-SCS/PMCG microcapsules; however, the composition of the gelling solution was altered. The gelling solution charged into the first reactor consisted of 20 mM BaCl_2_ dissolved in saline. The gelling solution charged into the second reactor contained 1.2 wt. % of RBITC-labeled PMCG dissolved in saline.

### Bright field optical microscopy

The size of prepared microspheres was determined using the optical microscope (Optika SRL, Ponteranica, Italy) equipped with a CCD camera (Motic Moticam 1SP 1.3MP) and software Motic Images Plus 2.0 (Kvant s.r.o., Bratislava, Slovakia). Determination of the microsphere size was performed for ca 20 microspheres from each batch and is reported as an averaged value ± standard deviation. Size was measured (in µm) as the diameter of the microspheres.

### Intraperitoneal implantation of microspheres in nude and C57bl/6 mice models

All animal procedures were approved by the Animal Care Committee at the University of Illinois at Chicago, and the methods were carried out in accordance with the relevant guidelines and regulations. During transplantation of the microspheres, the animals were anaesthetized using isoflurane and prepped for surgery. After surgical preparation of the incision site on the abdomen, a small 0.5 cm incision was performed to expose the intraperitoneal (IP) space. A sterile Pasteur pipette containing the microspheres to be transplanted in Hanks balanced salt solution containing 2 mM CaCl_2_ (approximately 0.4 cc of microspheres) was inserted into the IP space and the microspheres were evenly distributed throughout the IP space. The muscle tissue and skin were then sutured according to standard protocol and post-operative care was performed. Each microsphere type was implanted to three mice (n = 3).

### Explantation of microspheres from the intraperitoneal space of nude and C57bl/6 mice models

During explantation of the microspheres, the animals were euthanized in order to harvest all the microspheres and observe any effect in the IP space. The abdomen was prepared for incision as *per* implantation procedure. The microspheres were then retrieved using a sterile Pasteur pipette, and the IP space was washed with Hanks balanced salt solution containing 2 mM CaCl_2_ in order to explant all the freely suspended microspheres. Following this, the abdomen was fully exposed to explant any additional microspheres or clumps that were not removed through IP flushing. Prior to the CRM analysis, the explanted microspheres were stored in saline solution containing 2 mM CaCl_2_.

### Data availability

The datasets generated during and/or analyzed during the current study are available from the corresponding author on reasonable request.

## Results and Discussion

### CRM imaging of alginate microbeads: validation, technical aspects, and quantitative analysis

Alginate microbeads represent one of the most popular microsphere designs applied in the cell encapsulation field^1^, which can be ascribed to their well-documented immunoprotective character in various animal models and also to the overall simplicity of this one-component system. The preparation method allows for certain design flexibility, i.e., the localization of alginate in the microbeads can be influenced by the experimental conditions. The currently available microbead designs range from completely homogeneous ones to microbeads with a high degree of heterogeneity where alginate is concentrated in the outermost layer of the microbead. In this work, alginate microbeads were used as the main model microsphere for tailoring the CRM methodology to the needs of the microencapsulation field. Besides the acquisition of both qualitative and quantitative data on the spatial distribution of alginate, this included also validation of the method and addressing various technical aspects, such as fixing the position of the microbead during the analysis, verification of the calibration method, data processing (normalization and deconvolution of probing confocal volume), and choosing the optimal laser wavelength.

The workflow diagram in Supplementary Fig. S2 depicts the individual steps involved in the CRM data acquisition and processing. To localize alginate in the microbeads, the symmetric stretching band ν(COO^−^) of both free and complexed carboxylate residues at about 1415 cm^−1^ was selected in the Raman spectrum of sodium alginate (provided in Supplementary Fig. S4) because its intensity does not depend strongly on the ratio of mannuronic to guluronic acid residues^34^. The position of this band was reported to shift to higher wavenumbers by up to 20 cm^−1^ depending on the Ca^2+^ concentration used to form the alginate hydrogel^35,36^. In this work, this band shifted less significantly, up to about 10 cm^−1^ (Supplementary Fig. S10). In practical terms, CRM imaging is complicated by the necessity to maintain a constant position of microspheres during data acquisition that can take up to 60 minutes. We solved this problem by designing a custom holder, manufactured through the laser ablation technique, featuring bowl-shaped wells to accommodate individual microspheres (for details see Supplementary Fig. S3).

In order to validate the CRM data, CLSM was employed as it is currently the standard method for visualizing the spatial distribution of polymeric components in hydrogel microspheres used for cell encapsulation^18,20,37,38^. As mentioned above, CLSM requires sample labeling. This was achieved through the reaction of alginate with fluorescein amine. Subsequently, the labeled alginate was used for preparation of two types of alginate microbeads, i.e., homogeneous and heterogeneous, that were then analyzed by both CLSM and CRM to obtain relative concentration profiles of alginate (Fig. 1c,g). The obtained profiles overlap, which confirms that the CRM method provides results comparable to CLSM in the qualitative analysis.

Both CLSM and CRM analyses of hydrogel microspheres are affected by a certain error originating from the changes in signal attenuation as the laser beam passes through different regions of the microsphere during the scan along the equatorial microbead cross-section (the x-axis). The signal attenuation is caused mainly by energy absorption when the laser beam propagates through hydrogel layers of different density (different local concentration of polymers) and thickness. In CRM, however, these fluctuations can be compensated by normalization of the Raman signal intensity to the signal intensity of water^24^. The bending band of water at around 1640 cm^−1^ was selected for the normalization because it is insignificantly sensitive to factors influencing the hydrogen bonding interactions (e.g., temperature and ionic strength)^39^. This method results in a more accurate representation of polymer spatial distribution as compared to using the fluorescence signal in CLSM where the signal intensity loss cannot be compensated. In addition, corrections related to the deconvolution of the probing confocal volume can be applied in the CRM analysis. In Supplementary Fig. S9, this approach is demonstrated and the CRM and CLSM methods are contrasted using radial intensity profiles (a scan along the z-axis) of a highly heterogeneous alginate microbead where, due to the high hydrogel density in the outer regions, the differences in signal attenuation are expected to be most significant.

The relative concentration profiles represent useful information that can be correlated with the conditions used for microbead preparation. Nevertheless, especially with regard to transplantation of encapsulated cells, there is critical need for data on the absolute local polymer concentration in hydrogel microspheres. We therefore constructed calibration curves (Supplementary Fig. S7) and used them to transform the relative Raman intensity profiles into absolute alginate concentration profiles. As mentioned by Heinemann *et al*.^24^, preparation of homogeneous alginate microbeads of precisely defined alginate concentration is not experimentally feasible. This is mainly due to the unpredictable swelling or shrinking of the microbeads, which changes the local alginate concentration, and also because of technical reasons such as the very high viscosity of more concentrated alginate solutions. For this reason, we also resorted to using alginate solutions for calibration purposes. In the concentration range where the experimental reasons permitted, we successfully validated this approach by CRM analysis of homogeneous alginate hydrogel slabs with defined alginate concentration that were prepared by the internal gelling method^31^. The real concentration of alginate in the slabs was quantified by following their swelling kinetics (Supplementary Table S1). As seen in Supplementary Fig. S8, these values were within the predicted concentration interval determined by the quantitative CRM analysis.

The quantitative CRM analysis of the heterogeneous microbeads introduced above showed that the alginate concentration was about 12 wt.% at the surface, decreasing steeply to 0.5–1 wt.% in the microbead core (Fig. 1d). Figure 1h reveals that the absolute alginate concentration across the homogeneous microbeads was approximately 2 wt.%. Knowledge of the local alginate concentration in various alginate microbead designs is crucial as previous studies have confirmed that the fate of immobilized cells, characterized by their metabolic and secretory activities^13,40^, survival^41^, and differentiation^42^, strongly depends on the alginate concentration in the cell environment. For instance, Stabler *et al*. showed that the increase in concentration of high G alginate in solution used for preparation of microbeads from 1 to 2 wt.% causes significant retardation of metabolic and secretory activities in immobilized βTC3 cells due to the inhibition of their growth^40^. Similarly, BHK cells were shown not to survive in an alginate hydrogel made of 2 wt.% high G alginate^41^. However, due to the lack of suitable analytical methods, such studies correlated the cell performance only with nominal concentrations of alginate in the original solutions and not with true concentrations in the immediate proximity of the cells. Quantitative mapping of local alginate concentration offered by CRM can provide considerably more accurate data and help thus avoid conditions in preparation of microbeads that can lead to temporary or permanent deterioration of viability and functionality of encapsulated cells. On a general note, CRM can also bring the necessary insight into the dynamics of the crosslinked network formation and subsequent re-arrangement triggered by external stimuli in alginate-based hydrogels and similar materials.

To confirm that the quantitative CRM analysis is not influenced by the choice of the laser type, we analyzed selected microbeads using two different laser wavelengths, 532 nm and 785 nm. As shown in Supplementary Fig. S1, for the particular hydrogel microbeads tested here, comparable concentration profiles were obtained at both the wavelengths. In general, the 532 nm wavelength offers higher spatial resolution and is potentially less damaging to sensitive samples as lower average probing power can be used. However, we opted for the 785 nm wavelength as it is less susceptible to autofluorescence, which might be significant when analyzing explanted microspheres where protein adsorption cannot be ruled out. The CRM data presented in this paper were thus acquired using the 785 nm laser.

### Impact of environment on microsphere structure

Perhaps the most important area where CRM imaging can be applied is the visualization of changes to polymer spatial distribution in non-covalently crosslinked microspheres upon their exposure to different environments, especially *in vivo* conditions. As was previously mentioned, immunoprotective properties of the hydrogel microsphere are closely linked to the localization of polymeric components in the microsphere volume. Major changes to the polymer spatial distribution can thus compromise the protection that the microspheres provide to the encapsulated cells. For this reason, we employed CRM to determine how the structure of hydrogel microspheres responds to the changes of the environment. Two mainstream microsphere designs were studied: (i) heterogeneous, single-component, ionotropically gelled alginate microbeads, and (ii) three-component, polyelectrolyte complex-based microcapsules. In the following text, results obtained for each of the microsphere type will be discussed separately, which will form a basis for the subsequent comparison of the two designs.

Alginate microbeads used for immunoprotection of transplanted cells have been intentionally prepared with either higher^5^ or lower^6,15^ degree of heterogeneity. It is generally assumed that the immunoprotective properties are related to the localization of alginate in the microbead^13^. However, whether the initial spatial distribution of alginate is maintained when the microbeads are exposed to different environments, including the *in vivo* environment, remains currently unknown. Using preparation conditions similar to those previously utilized in clinical trials^5,6^, two types of heterogeneous alginate microbeads were prepared with markedly different initial degrees of heterogeneity. The preparation and storage medium was a D-mannitol solution, which ensured preservation of the microbead structure because anti-gelling sodium ions were absent. Before implantation, the microbeads were transferred to saline. In order to achieve more representative comparison of microbeads before implantation and after explantation, a small fraction of these microbeads was separated and incubated for 24 hours at 37 °C to simulate the potential body temperature influence. The remaining microbeads were implanted into the peritoneal cavity of nude mice for a period of 4 weeks, and stored in saline after explantation. To evaluate the effects of environment, the microbeads were analyzed by CRM after the three different stages: preparation (mannitol storage), incubation in saline at 37 °C for 24 h, and explantation (Fig. 2). Note that CaCl_2_ was added to all the storage media (2 mM final concentration) to mimic the physiological concentration of Ca^2+^ ions. This is of particular relevance in the case of explanted microbeads where CaCl_2_ helps conserve the attained alginate concentration profile prior to the CRM analysis.

Optical microscopy images of the more heterogeneous microbeads shortly after preparation reveal that the used gelling conditions afforded the expected core/shell morphology resulting from the gelling front movement from the microbead surface towards its core (Fig. 2a)^29^. A shell of about 40 μm thickness was formed, corresponding to the local alginate concentration of about 10 to 11 wt.%. This shell could be identified by both optical microscopy and CRM. Closer to the microbead core, CRM alone was capable of visualizing the regions of different local alginate concentration that reached a minimum of about 0.5 wt.% in the microbead core. Figure 2b depicts the microbeads incubated for 24 h at 37 °C in saline and indicates that the level of heterogeneity was significantly reduced compared to microbeads stored in D-mannitol, with a maximum alginate concentration at the microbead surface of about 6 wt.% and about 1 wt.% in the core. Such a change is not surprising and is ascribed to the influence of the non-gelling sodium ions causing a partial dissolution of the alginate hydrogel network due to the exchange of the crosslinking ions for non-gelling sodium ions^20^. The attained profile did not alter with time, indicating that Fig. 2b represents the equilibrium spatial distribution of alginate in a saline environment. Strikingly, in the microbeads exposed to the *in vivo* environment, the heterogeneity of alginate spatial distribution diminished almost completely, i.e., only minor differences in alginate concentration were observed, with up to 4 wt.% at the surface and 2–3 wt.% across the rest of the microbead (Fig. 2c).

The CRM profiles and optical microscopy images of alginate microbeads prepared with a lower degree of heterogeneity are shown in Fig. 2d–f. These microbeads were treated in the same way as the more heterogeneous microbeads above. While the optical microscopy images are virtually identical in the respective stages, the CRM profiles reveal a different picture, i.e., changing levels of heterogeneity. The span of alginate concentrations between the microbead surface and core was approximately 6 to 0.5 wt.%, 3.5 to 1.0 wt.%, and 3.0 to 1.5 wt.% for microbeads after preparation (D-mannitol storage), after 24 hours storage at 37 °C in saline, and after explantation, respectively. Notably, the final profiles for both the analyzed microbead designs, shown in Fig. 2c,f, are visually similar, confirming that the ultimate alginate spatial distribution in the microbeads exposed to the *in vivo* environment does not depend significantly on the used gelling conditions (i.e., the initial microbead heterogeneity). Note that the gelling process causes shrinking of the original alginate droplets, concentrating alginate in the resulting microbeads. This shrinkage is different for the two gelling methods used here, which explains the concentration and size differences visible for the explanted microbeads (Fig. 2c,f).

The second type of microspheres whose structure was followed by the CRM imaging were multi-component hydrogel microcapsules, illustrating the utility of the CRM methodology in the analysis of complex systems^43^. Specifically, we employed a microcapsule made of three polymeric components: sodium alginate (SA), sodium cellulose sulfate (SCS) and poly(methylene-*co*-cyanoguanidine) (PMCG)^26^. Encapsulation of islets of Langerhans in this microcapsule has led to diabetes reversal in various animal models^26,44,45^, and recently showed biotolerability in a preclinical model of non-human primates^46,47^. The microcapsule is predominantly stabilized by two coexisting networks: (1) an ionotropic network between calcium or barium cations and alginate, and (2) a polyelectrolyte complex between SCS and PMCG^26^. The microcapsules were analyzed by CRM at two stages: prior to implantation (storage in saline) and after explantation from the intraperitoneal cavity of C57bl/6 mice 2 weeks post-implantation (Fig. 3) or nude mice 4 weeks post-implantation (Supplementary Fig. S15). Note that the microcapsule depicted in Fig. 3 was made using D-mannitol as an osmolyte in the first step of the microcapsule preparation, i.e., under conditions similar to those used for the preparation of alginate microbeads shown in Fig. 2 above. We see this as the key point for the side-by-side comparison of the two encapsulation designs. The following Raman bands were used for determining the spatial distribution of the individual polymeric components: 1415 rel. cm^−1^ for SA, 1070 rel. cm^−1^ for SCS, and 770 rel. cm^−1^ for PMCG (Supplementary Fig. S6). Validity of the obtained data was confirmed by analyzing microcapsules made of fluorescently labeled PMCG by both CRM and CLSM (Supplementary Fig. S14). In contrast to the alginate microbeads discussed above, data on absolute concentrations of the individual polymers cannot be easily extracted in the present multi-component system. Nevertheless, relative changes in the spatial distribution of the individual polymeric components can be assessed. CRM imaging can thus bring useful insight into the mechanism of hydrogel network formation and provide information on how the microcapsule structure responds to the changing environment.

Figure 3 shows the optical microscopy images of the studied microcapsules together with the obtained spatial distribution profiles for all the three polymeric components. The optical microscopy images imply that the SA-SCS/PMCG microcapsule consists of a core that is covered by a membrane of several tens of micrometers in thickness. In general, the CRM data show that the polymers are concentrated in the outermost region of the microcapsule. Inside the microcapsule, SA is the main polymeric component (around 35% relative concentration) while SCS and PMCG show only negligible presence here. In the microcapsule prior to the implantation, CRM reveals two distinct regions in the outer sphere (see the detailed Fig. 3c). We postulate that the outermost layer (ca 0–40 µm from the surface) roughly overlaps with the visually observable membrane. SA co-localizes with PMCG at the membrane and its relative concentration is highest here. The second layer (between ca 40 and 60 µm from the surface) appears to represent the outermost region of the core. Here, PMCG co-localizes with SCS, with both the polymers showing their concentration maxima. This co-localization indicates the formation of a strong polyelectrolyte complex between SCS and PMCG^26^, which agrees qualitatively with the data obtained previously by CLSM^38^. The two layers are separated with a region where all the three polymers show their local concentration minima. The microcapsule structure observed by CRM reflects well the main processes taking place during the 2-step microcapsule preparation. In the first step, a microbead stabilized predominantly by an alginate network is formed that contains trapped SCS. In the second step, the microbead is exposed to cationic PMCG, which results in the membrane formation. PMCG then diffuses further towards the core, but this process is apparently progressively slowed down by complexation with SCS. In this way, another SCS-PMCG polymer-rich region is formed, i.e., the second layer observed by CRM. Limited diffusion of high-molecular weight fractions of SCS throughout the hydrogel network from the core towards the microcapsule surface can also account for this observation.

In the explanted microcapsule (Fig. 3f), a simpler concentration profile was observed with all three polymeric components showing their relative concentration maxima in the outermost layer of the microcapsule, i.e., the membrane. The second layer became much less prominent as illustrated by only small local maxima of SCS and PMCG relative concentrations. A slight shift (ca 10 µm) of this layer from the surface towards the microcapsule center was observed. Apparently, the *in vivo* environment also caused certain transfer of SA from the core towards the membrane, which is visualized by the decrease in its relative concentration in the core from about 35% to about 25%. Taking into account the considerable polymer mass transfer observed in alginate microbeads above, it is possible that the loosening of the alginate/divalent ions network in the *in vivo* environment accounts for the alginate mobility and also allows the spatial rearrangement of the remaining two polymers, especially the high-molecular weight SCS. The  increase in the apparent heterogeneity in the alginate distribution profile can then be ascribed to the SA-PMCG interactions that, despite being weaker than SCS-PMCG interactions^26^, can still influence mobility of SA. The data qualitatively identical to those shown in Fig. 3 were obtained for SA-SCS/PMCG microcapsules prepared at slightly different gelling conditions using saline instead of D-mannitol in the first gelling step (Supplementary Fig. S15). These conditions led to slightly lower heterogeneity of the microcapsule structure that was maintained *in vivo*. In this case, SA-SCS/PMCG microcapsules were implanted intraperitoneally to nude mice and explanted 4 weeks post-implantation, i.e., the identical *in vivo* conditions were used as for alginate microbeads (Fig. 2).

The obtained CRM data show that alginate microbeads and multi-component microcapsules have considerably different structural stability in the *in vivo* environment. In general, both alginate microbeads and alginate-based microcapsules are composed of non-covalently crosslinked hydrogel networks stabilized predominantly by electrostatic interactions^48^. The number and position of crosslinks can fluctuate in response to environmental characteristics such as osmotic pressure and presence of ions and other molecules (e.g., proteins in the *in vivo* environment). For both the heterogeneous alginate microbead designs studied here, the heterogeneous character decays during storage in saline, reaching an equilibrium profile with certain heterogeneity retained, and almost completely diminishes when the microbeads are exposed to the *in vivo* environment. At present, it is not clear which characteristics or components of the *in vivo* environment are responsible for the observed partial dissolution and equilibration of the ionotropically stabilized alginate network. Nevertheless, the obtained data allow us to hypothesize that in all the previously performed *in vivo* studies involving alginate microbeads, the spatial distribution of alginate eventually became homogeneous regardless of the preparation conditions. From this point of view, it appears futile to attempt at specific control of the initial heterogeneity of alginate microbeads. Furthermore, it can be expected that the loss of microbead heterogeneity upon exposure to the *in vivo* environment leads to an increase in the molecular weight cut-off (MWCO) of the microbeads, possibly deteriorating immunoprotective properties of the related cell encapsulation systems. Since considerable loss of heterogeneity was observed even in the saline environment, it is not surprising that the previously reported MWCO values for alginate microbeads of vastly different (initial) heterogeneity, exposed to saline after preparation, were similar (250 to 350 kDa, protein equivalent)^15,49^. It follows that the heterogeneous character of alginate spatial distribution has to be conserved by means other than ionotropic gelling, e.g., by polyelectrolyte complexation with a polycation^11,20^ or by a combination of polyelectrolyte complexation with covalent crosslinking^50,51^. This view is supported by our CRM results on structural changes in the SA-SCS/PMCG microcapsules. In this case, partial dissolution of the ionotropic alginate network enabled post-complexation processes accompanied by migration of SCS and PMCG towards the microcapsule membrane, conserving thus the heterogeneous profile of the microcapsules. Consequently, from the structural point of view, SA-SCS/PMCG microcapsules qualify for a better control of immunoprotective properties than alginate microbeads.

## Conclusions

In summary, this work establishes CRM imaging as a non-invasive method to provide quantitative and/or qualitative data on the spatial distribution of polymers in non-covalently crosslinked hydrogel microspheres used for immunoprotection of transplanted cells. To achieve this, various experimental aspects were addressed, including CRM data validation by CLSM, construction of calibration curves for quantitative analysis, verification of the quantitative data, comparison of different laser wavelengths, or fixing the position of microspheres during their CRM analysis. Through the quantitative mode of CRM imaging, the true alginate concentration was mapped throughout the whole volume of alginate microbeads of different heterogeneity, providing essential information about the environment that cells will encounter upon their encapsulation. Even more importantly, CRM imaging was shown to be a powerful tool in visualizing the structural changes in microspheres, triggered by the changing environment. It was found that the two model microsphere types used here differ considerably in their structural stability in the *in vivo* environment, which may impact their immunoprotective properties. While the heterogeneous alginate microbeads lost their heterogeneous character upon implantation, the multi-component microcapsules largely retained their structure at the same conditions. The final alginate microbead structure was found to be largely independent on the initial degree of microbead heterogeneity, so it appears unnecessary to attempt at control of this parameter when *in vivo* applications are targeted. In conclusion, the CRM method can facilitate the assessment of immunoprotective properties of different microsphere designs and streamline thus the development of new cell encapsulation systems. The presented results illustrate that CRM has the potential to become a preferred method in laboratories involved in cell encapsulation and other related fields, effectively replacing CLSM. Especially for multi-component systems, there appears to be no alternative to CRM imaging.

## Electronic supplementary material

Supplementary information
