# Supplementary material for: Structural changes in alginate-based microspheres exposed to in vivo environment as revealed by confocal Raman microscopy
Source: Sci Rep. 2018 Jan 26;8:1637. doi: 10.1038/s41598-018-20022-y (PMC5785987; doi:10.1038/s41598-018-20022-y)
Supplement: Supplementary file 1 — Supplementary information [file 41598_2018_20022_MOESM1_ESM.pdf]

Supplementary information

Structural changes in alginate-based microspheres exposed to *in vivo* environment as revealed by confocal Raman microscopy

Zuzana Kroneková,[1#] Michal Pelach,[2#] Petra Mazancová,[1#] Lucia Uhelská,[1] Dušana Treľová,[1] Filip Rázga,[1] Veronika Némethová,[1] Szabolcs Szalai,[1] Dušan Chorvát,[4] James J. McGarrigle,[5] Mustafa Omami,[5] Douglas Isa,[5] Sofia Ghani,[5] Eva Majková,[2] José Oberholzer,[5] Vladimír Raus,[1,3] Peter Šiffalovič,[2#] Igor Lacík[1]*

[1]Department for Biomaterials Research, Polymer Institute of the Slovak Academy of Sciences, Dúbravská cesta 9, 845 41 Bratislava, Slovakia

[2]Department of Multilayers and Nanostructures, Institute of Physics of the Slovak Academy of Sciences, Dúbravská cesta 9, 845 11 Bratislava, Slovakia

[3]Institute of Macromolecular Chemistry, Academy of Sciences of the Czech Republic, Heyrovsky Sq. 2, 162 06 Prague 6, Czech Republic

[4]Department of Biophotonics, International Laser Center, Ilkovicova 3, 841 04 Bratislava, Slovakia

[5]Division of Transplantation, Department of Surgery, University of Illinois at Chicago, 840 South Wood Street, Chicago, Illinois 60612, USA

# equally contributing authors

* corresponding author (email: igor.lacik@savba.sk, ph. +421 903789107)

Figure S1: Raman signal obtained by CRM imaging of alginate microbeads at the laser lines 532 and 785 nm.

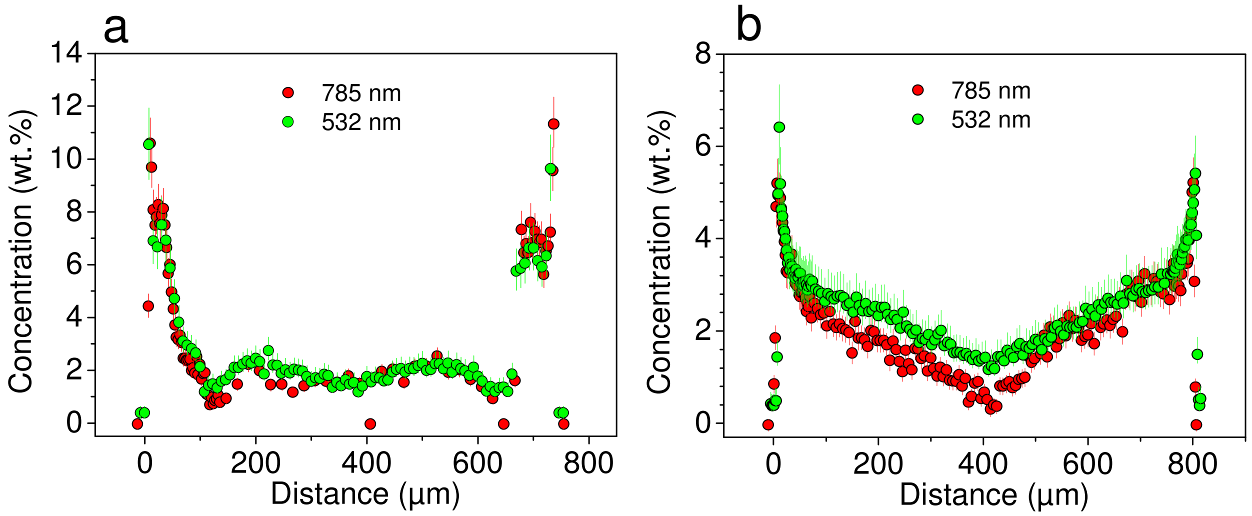

(**a**) Concentration profiles of alginate determined by CRM for alginate microbeads of higher heterogeneity after preparation (stored in D-mannitol). (**b**) Concentration profiles of alginate determined by CRM for alginate microbeads of lower heterogeneity after preparation (stored in D-mannitol). The calibration curves for calculation of the absolute alginate concentration profiles from Raman signal intensities using laser lines 785 and 532 nm are shown in **Fig. S7a** and **S7b**, respectively.

Figure S2: Work flow diagram of CRM imaging of microspheres

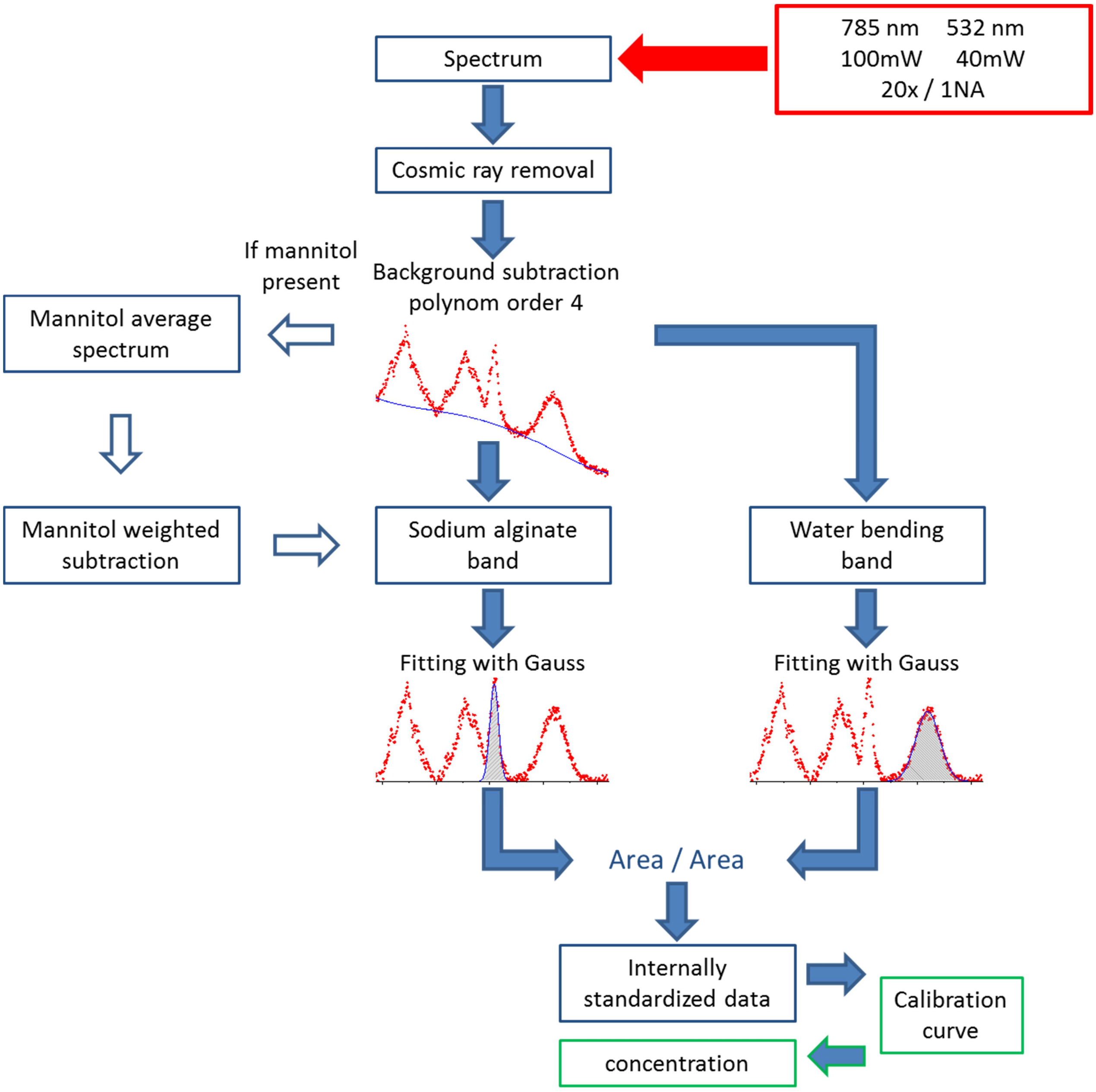

CRM imaging of hydrogel microspheres is exemplified for alginate microbeads. The step-by-step protocol involves spectrum acquisition, removal of cosmic rays, background subtraction, fitting the sodium alginate stretching band and the water bending band, internal standardization of data, application of the calibration curve, and extraction of an absolute alginate concentration.

Figure S3: The custom-designed holder for CRM imaging of microspheres and the mode of line-scan

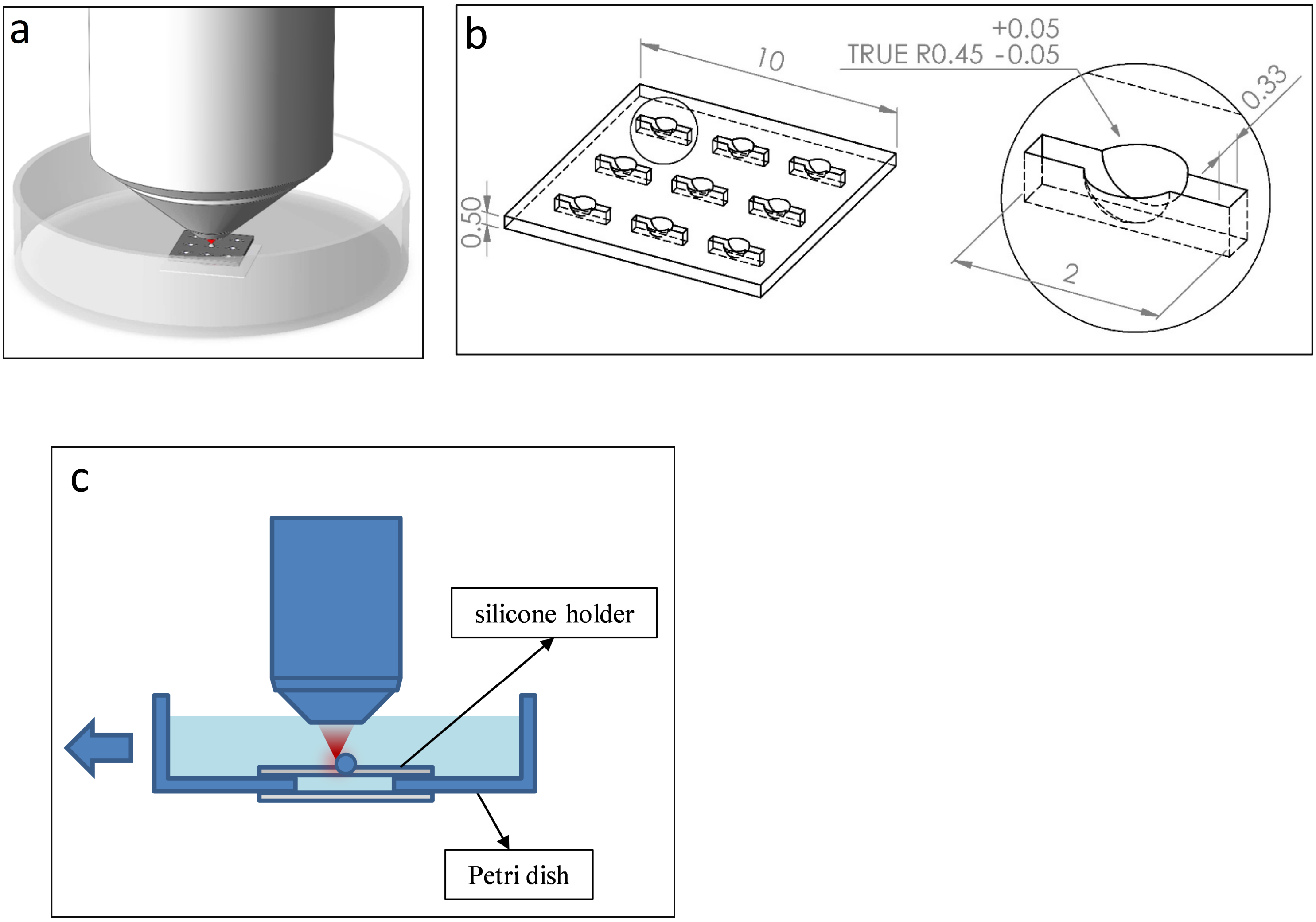

(**a**) The setup for CRM imaging of hydrogel microspheres in a liquid environment including the silicone holder mounted in a Petri dish under the objective. (**b**) Technical drawing of the holder used to maintain a constant position of microspheres during imaging (dimensions are in mm). The holder was made of silicone by the laser ablation technique. The bowl-shaped wells have diameters between 800 and 1000 µm and feature rectangular openings to prevent interference of the laser beam with the holder. (**c**) Side view of the setup depicting the microscope cover slip (thickness 170 µm) that is bonded by an epoxy resin to the bottom of the polystyrene Petri dish to cover the opening fabricated by laser ablation. This is done in order to prevent the interference of the back-scattered laser beam from the bottom of the Petri dish. The arrow shows movement of the stage to perform the line scan of a microsphere from left to right.

Figure S4: Raman spectrum of sodium alginate

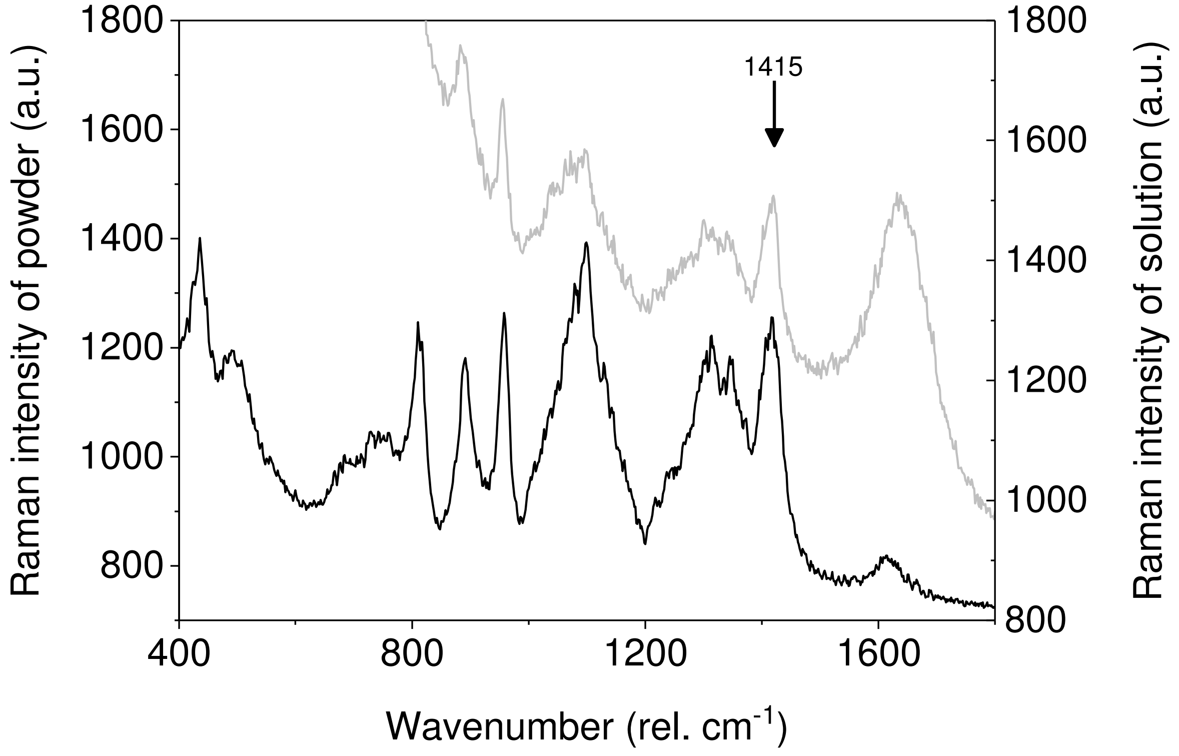

Raman spectrum of sodium alginate (SA) as powder (black line) and as 3 wt.% solution in saline (grey line). The arrow points at the selected band at 1415 rel. cm^{-1} associated with the symmetric vibration ν(COO^{-}).

Figure S5: Raman spectra of sodium cellulose sulfate and poly(methylene-co-cyanoguanidine)

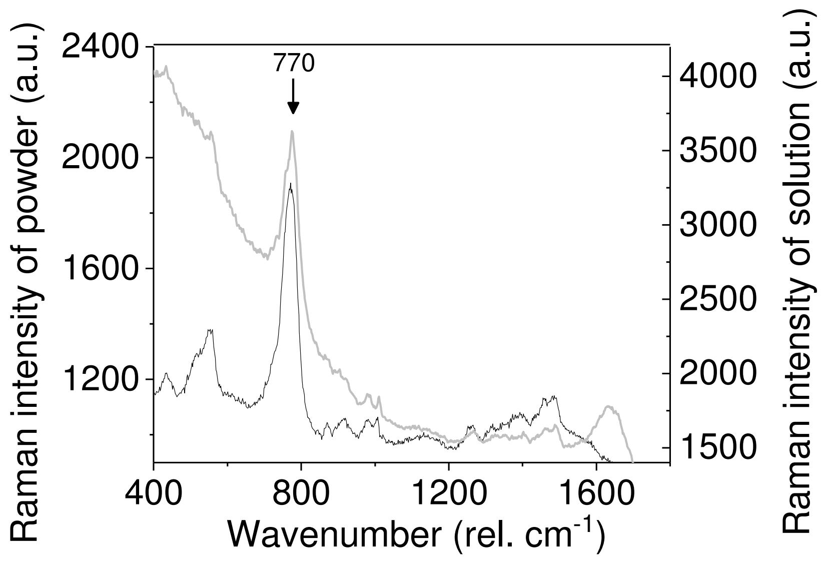

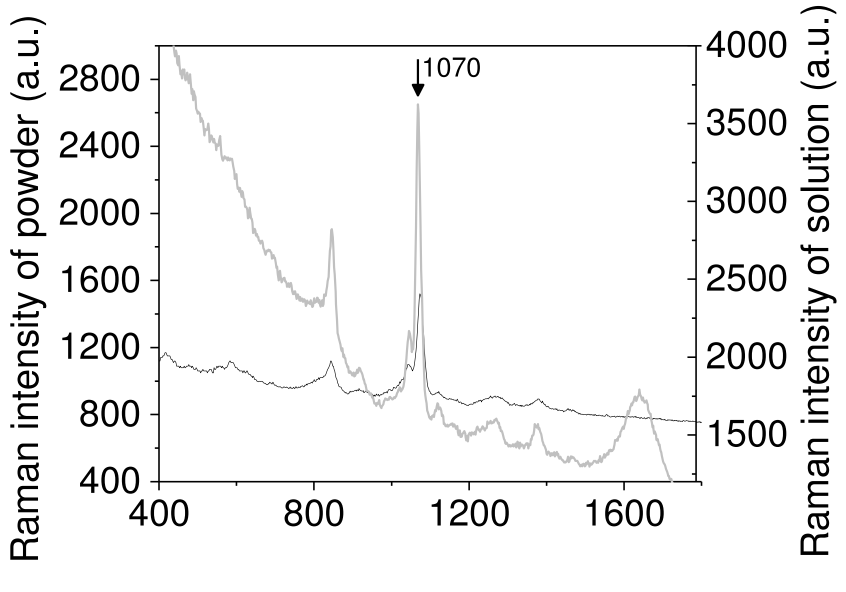

(**a**) Raman spectrum of poly(methylene-co-cyanoguanidine) as powder (black line) and as 3 wt.% saline solution (grey line). The arrow points at the selected band at 770 rel. cm^{-1}. (**b**) Raman spectrum of sodium cellulose sulfate powder (black line) and 3 wt.% saline solution (grey line). The arrow points at the selected band at 1070 rel. cm^{-1} associated with the symmetric stretching vibration of ν_s(O=S=O).

Figure S6: Raman spectrum of a multicomponent microcapsule made of sodium alginate, sodium cellulose sulfate and poly(methylene-co-cyanoguanidine)

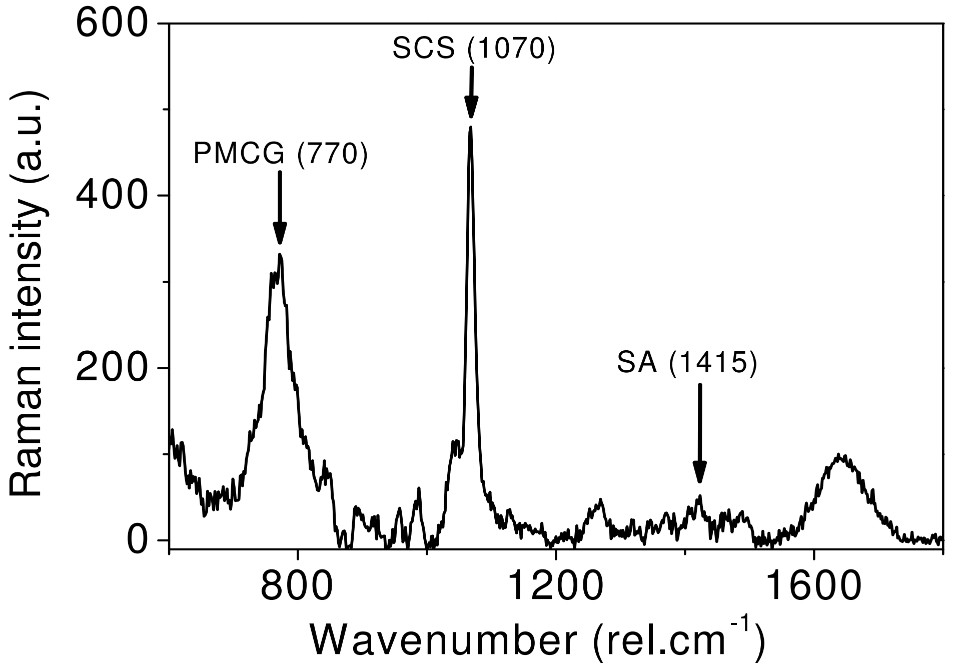

Raman spectrum of the multicomponent microcapsule made by polyelectrolyte complexation of sodium alginate (SA), sodium cellulose sulfate (SCS) and poly(methylene-co-cyanoguanidine) (PMCG). The arrows and wavenumbers indicate the selected characteristic bands of the respective polymers.

Figure S7: Calibration curves for determination of the absolute concentration of alginate in alginate microbeads from the Raman signal.

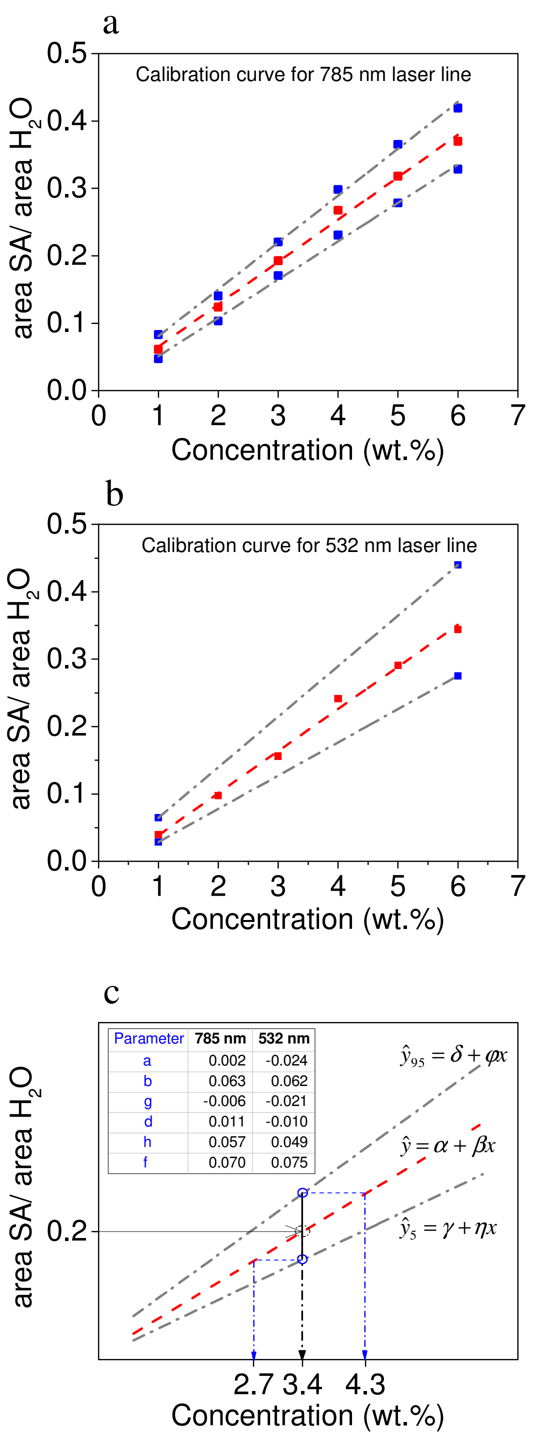

The absolute alginate concentration was calculated from the ratio of the respective alginate (area SA) and water (area H_2O) bands plotted as a function of SA concentration in saline solution. (**a-d**) Red squares are medians; blue squares are positions of 95 and 5 percentiles. Red dashed lines are linear fits of medians. Grey dash dot lines are linear fits of percentiles. (**a**) Calibration curve obtained for the 785 nm laser line (concentration range from 1 to 6 wt.%). (**b**) Calibration curve obtained for the 532 nm laser line (concentration range from 1 to 6 wt.%). (**c**) Retrieval of relevant concentration values with upper and lower limits $\hat{x}_{95}$ and $\hat{x}_5$ expressing the error bar, exemplified for the SA concentration

of 3.5 wt.%. Table in the inset shows the parameters for linear fits of medians and 95 and 5 percentiles for both laser lines. The calibration solutions of a high alginate concentration (> 3 wt.%) were made with alginate of molecular weight decreased by sonication.

Figure S8: Concentration of alginate in planar alginate hydrogel slabs.

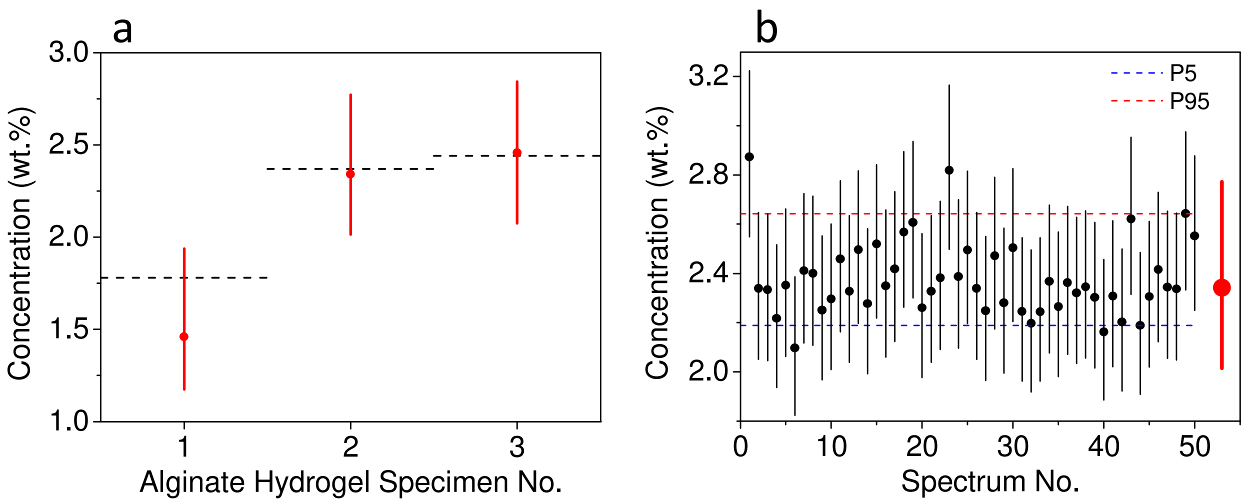

Alginate concentration in planar alginate hydrogels with homogeneous distribution of alginate of known concentration (**Table S1**), determined using the calibration curve based on alginate solutions (**Fig. S7**). (**a**) Three planar alginate hydrogel slabs were analyzed. Dashed lines are true concentrations of alginate, and points with error bars are concentrations determined using CRM imaging and calibration curve (laser line 785 nm). (**b**) Determination of alginate concentration from individual Raman spectra for the hydrogel specimen No. 2 as median of the measured data. The spectra were collected from the areas of 1 x 1 mm^2 and 0.1 x 0.1 mm^2 with no significant differences among collected spectra, which shows that the prepared hydrogels are homogeneous with respect to the alginate concentration.

Figure S9: Comparison of the radial intensity profiles obtained by CLSM and CRM when scanning a highly heterogeneous alginate microbead along the z-axis.

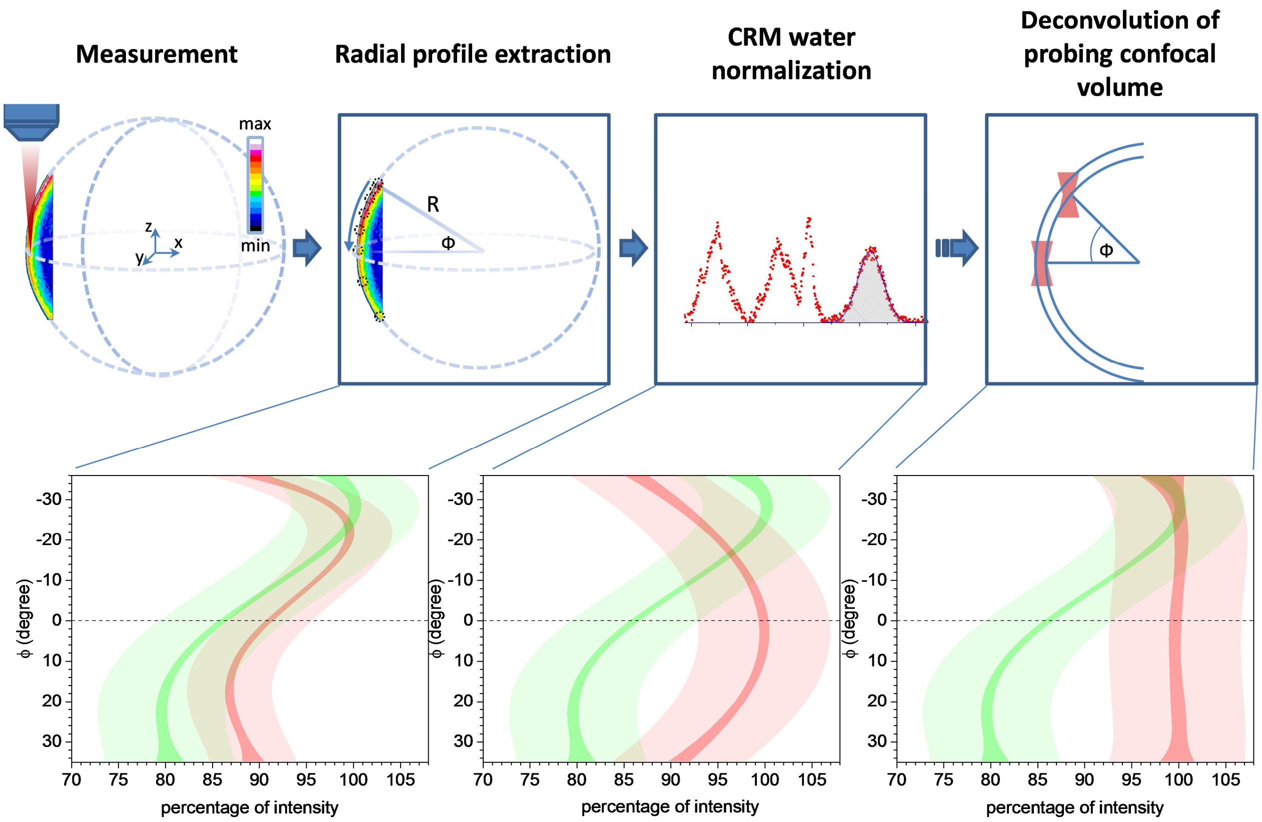

Upper row: schematic depiction of the individual steps of the data processing workflow. Bottom row: the corresponding radial intensity profiles represented by concatenated fits of multiple measurements (CLSM in green, CRM in red, prediction bands in pale colors, confidence bands in darker colors). The workflow starts with the CRM or CLSM analysis of the microbead by scanning along the z-axis, focusing at the outermost, high-alginate concentration, interfacial region (constant R in polar coordinates), which is followed by extraction of the signal intensity values and adjustment of the CRM and CLSM intensity profiles for a common scale. Further, normalization to the Raman band of water and readjustment of the signal for a common scale are performed for the CRM data. Finally, deconvolution of the varying spatial overlap between the probing CRM volume and the interfacial, high-alginate concentration region of the microbead is carried out for the normalized CRM signal.

Radial profiles in polar coordinates are used here to illustrate the variable CRM and CLSM signal attenuation resulting from the fact that, when performing the scan along the z-axis of the microbead, the incident laser beam has to travel through regions of different thickness and different alginate concentration. As can be seen, CLSM and CRM show a comparable signal loss pattern, which is reflected in very similar, significantly distorted, S-shaped radial profiles (bottom left). Multiple effects, including light absorption, varying spatial overlap of the probing volume with the inspected interfacial layer, and refraction effects, have an impact on the observed deviations of the profiles. For the CRM signal, a two-step correction can be applied to compensate these effects. In the first (key)

step, normalization using the Raman band of water effectively removes the absorption effects, which results in a C-shaped radial intensity profile (bottom center). This shape stems from the changing spatial overlap of the CRM confocal volume with the high-alginate concentration, interfacial layer of the microbead. In the second (optional) step, deconvolution is applied to account for this problem, arriving to the expected constant signal intensity as shown by the flattened radial profile (bottom right). For the microbead analyzed here, the provided data show that, at the $\phi = 0$ default measuring depth (i.e., scanning in the x-axis used for CRM analyses in this work), the corrected CRM signal has zero intensity loss while the CLSM signal shows approximately 15% intensity loss (average values in both cases). Also note that at $\phi = 0$, the deconvolution step is not necessary as the loss of the normalized signal is negligible; therefore, deconvolution was not routinely performed for the CRM data presented in this work.

Figure S10: Raman spectrum obtained for an alginate microbead in D-mannitol solution

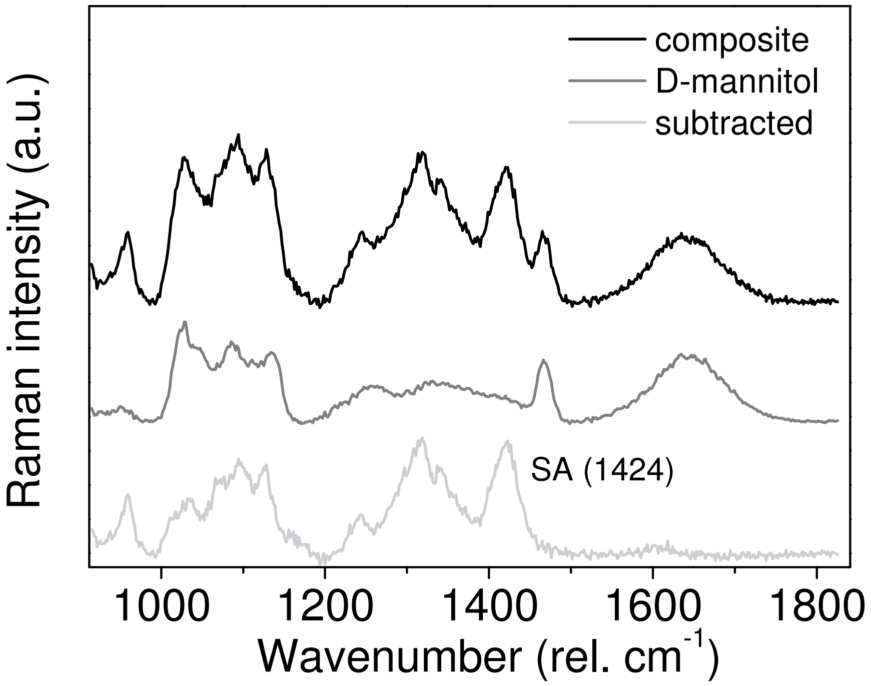

Some of the microspheres were measured in a 0.3 M D-mannitol solution. D-mannitol has several vibration bands which overlap with alginate vibrations. For this reason, the D-mannitol bands need to be removed from spectra in order to extract the alginate spectra. The fitting of D-mannitol bands is complicated because some bands are composite bands that are difficult to deconvolute. Therefore, weighted subtraction of D-mannitol spectrum was performed instead where this spectrum was measured in the solution outside of the microbead. Weighting was done using the water bending band. Subtracted spectrum was then fitted *via* the procedure already described. The water bending band was fitted before subtraction. The symmetric stretching band $\nu(COO^{-})$ for carboxylate residues was positioned at 1424 cm^{-1}.

Figure S11: Optical microscopy images of explanted alginate microbeads

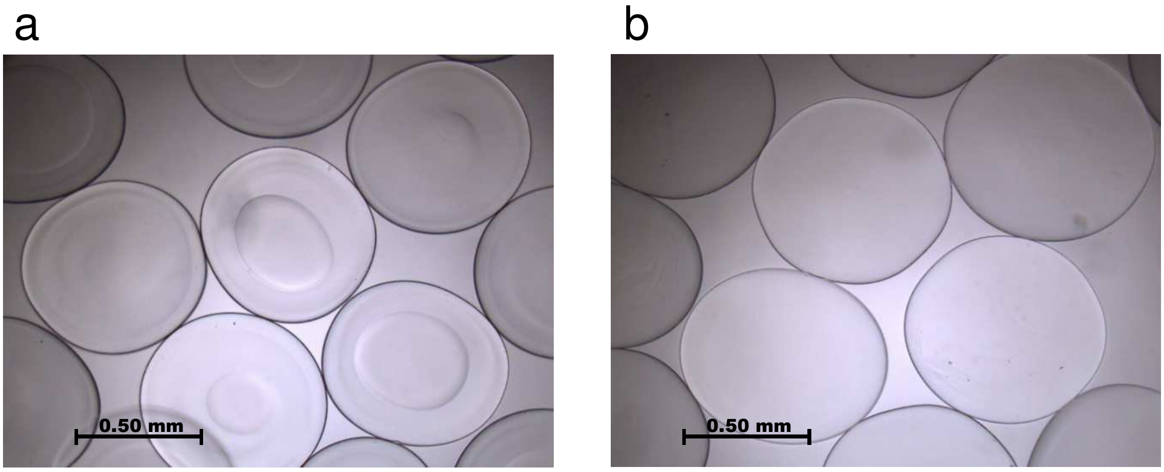

Examples of alginate microbeads explanted from the peritoneal cavity of nude mice 4 weeks post-implantation. (**a**) Microbeads of a higher degree of heterogeneity of spatial distribution of alginate. The core/shell morphology of a different degree can be noticed. (**b**) Microbeads of lower degree of heterogeneity of spatial distribution of alginate. These microbeads do not exhibit the core/shell morphology.

Figure S12: CRM concentration profiles and optical microscopy images for alginate microbeads of higher heterogeneity of alginate spatial distribution exposed to different environments

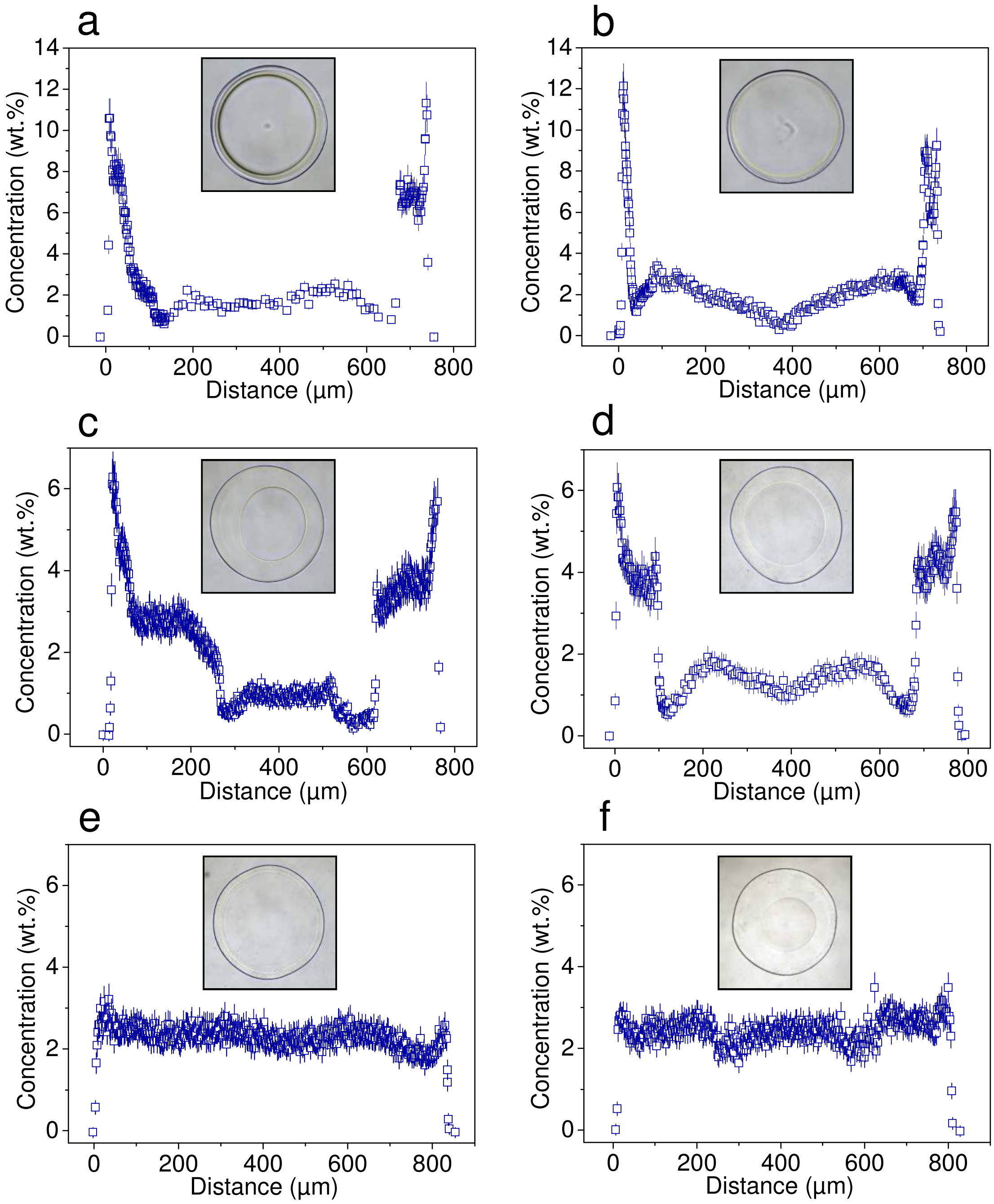

(**a,b**) Microbeads stored in 0.3 M D-mannitol solution. (**c,d**) Microbeads stored in saline at 37 °C for 24 h. (**e,f**) Microbeads explanted from nude mice 4 weeks post-implantation. Note various degrees of core/shell morphology for microbeads after preparation, after storage in saline, and after explantation. These are additional images to those shown in **Fig. 2a-c**.

Figure S13: CRM concentration profiles and optical microscopy images for alginate microbeads of lower heterogeneity of alginate spatial distribution exposed to different environments

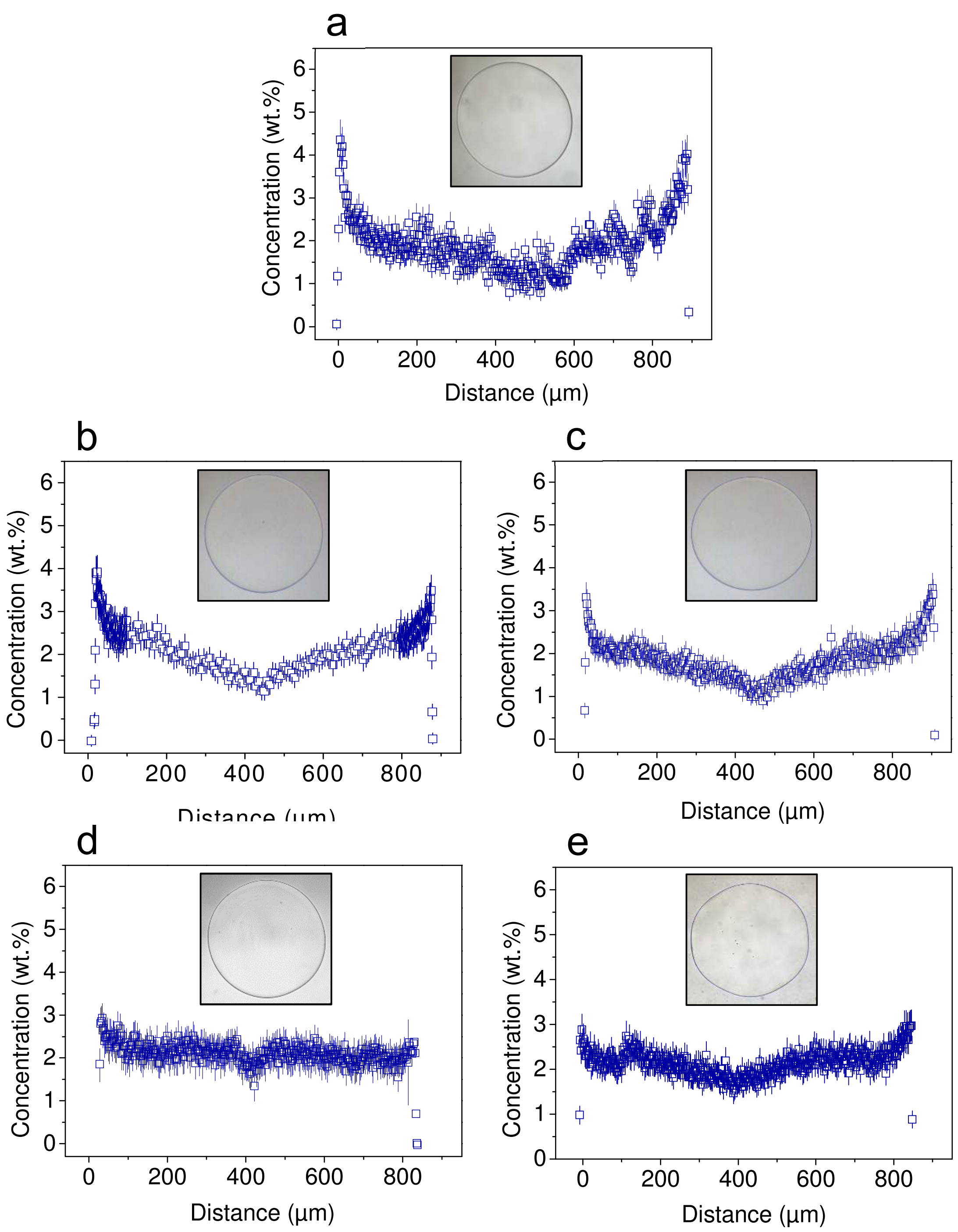

(**a**) Microbeads stored in 0.3 M D-mannitol solution. (**b,c**) Microbeads stored in saline at 37 °C for 24 h. (**d,e**) Microbeads explanted from nude mice 4 weeks post-implantation. These are additional images to those shown in **Fig. 2d-f**.

Figure S14: Validation of confocal Raman microscopy (CRM) by confocal laser scanning microscopy (CLSM) for multicomponent microcapsules made of sodium alginate, sodium cellulose sulfate and poly(methylene-*co*-cyanoguanidine)

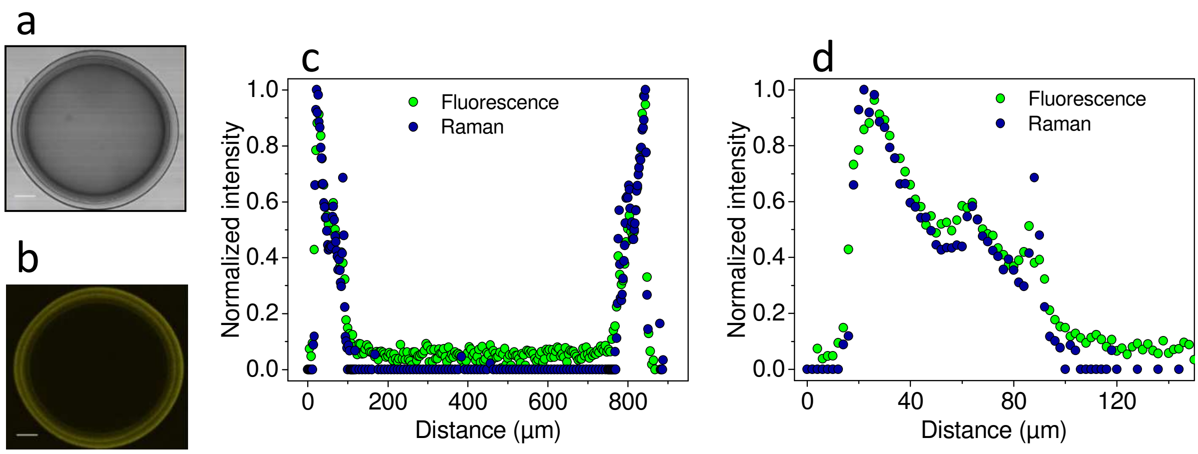

(**a-d**) Microcapsules prepared using rhodamine-labeled poly(methylene-*co*-cyanoguanidine). (**a**) CLSM image in the transmission mode. (**b**) CLSM image in the fluorescence emission mode at the equatorial microcapsule cross-section using λ_{exc} = 543 nm and a long-pass 560 nm emission filter. (**c,d**) The profile of spatial distribution of poly(methylene-*co*-cyanoguanidine) at the equatorial microcapsule cross-section for the entire microcapsule (**c**) and for the outermost region (membrane) (**d**) obtained by CRM (785 nm laser line) and CLSM imaging. The CRM and CLSM signal intensities are normalized to the intensity maxima. Bars (**a,b**) are equal to 100 µm.

Figure S15: Confocal Raman microscopy (CRM) of multicomponent microcapsules made of sodium alginate, sodium cellulose sulfate and poly(methylene-*co*-cyanoguanidine) before implantation and 4 weeks post-implantation from nude mice

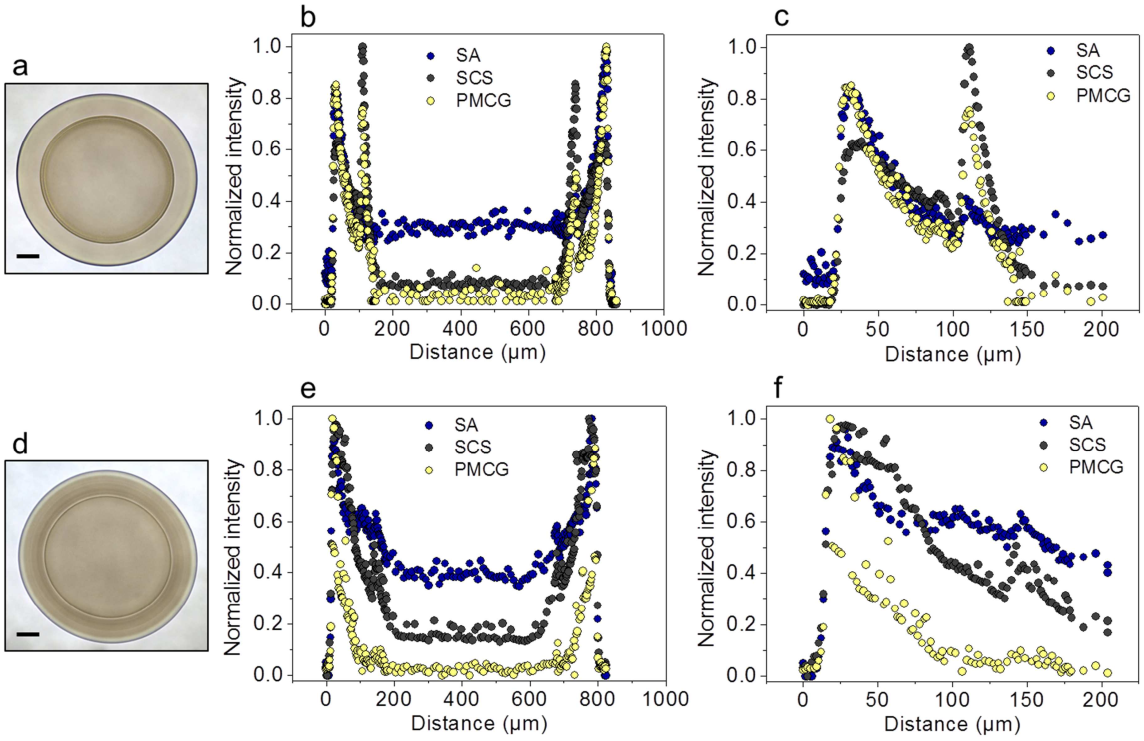

CRM intensity profiles (equatorial cross-section) of a multi-component microcapsule (SA/SCS-PMCG) after preparation (**a-c**), and after explantation from the intraperitoneal space of nude mice 4 weeks post-implantation (**d-f**). (**a, d**) Optical image (bar equals to 100 µm). Spatial distribution of individual polymeric components within the entire microcapsule (**b, e**) and in the outermost region only (**c, f**) obtained from Raman signal normalized to the maximum intensity.

Table S1: Steps in the preparation of planar alginate hydrogels of homogeneous spatial distribution of alginate by the internal gelling method. The starting concentration of sodium alginate solution, c_0(SA), is 2 wt.%.

Post-gelling solution[a)]	Weight of hydrogel slabs (g)				Weight change (%)			Final concentration of hydrogel (wt.%)[c)]
	Casted to the glass mold[b)]	After internal gelling	After 10 min post-gelling treatment	After 24 h in storing solution	After internal gelling	After 10 min post-gelling treatment	After 24 h in storing solution	
Saline	0.780	0.722	0.780	0.874	-7.4	0	+12.1	**1.78**
50 mM $CaCl_2$ in saline	0.670	0.626	0.490	0.566	-6.6	-26.9	-15.5	**2.37**
5 mM $CaCl_2$ in saline	0.810	0.771	0.812	0.665	-4.9	0	-17.9	**2.44**

a) Treatment of hydrogel slabs after the initial internal gelling step.

b) The reference weight to which the weight after each step is compared.

c) Determined as c_0(SA) / (1 + Δm/100), where c_0(SA) is the initial concentration of sodium alginate in solution (in wt.%) and Δm is the final weight change of hydrogel slabs after 24 h in storage solution (in %).

Supplementary Note 1: CRM data processing

The Project Four+ software from WITec GmbH was used for data processing. First, the cosmic rays removal was performed on acquired spectra, which helps to reduce artifacts in further data processing. Next, the background subtraction was performed. A polynomial of the 4th order was used for alginate microbeads. The regions where polynomial fitting was performed are shown in **Table SN1.1**.

Table SN1.1: Polynomial fitting for background subtraction in CRM imaging of alginate microbeads

	Region of polynomial fitting (rel. cm^{-1})			
	1	2	3	4
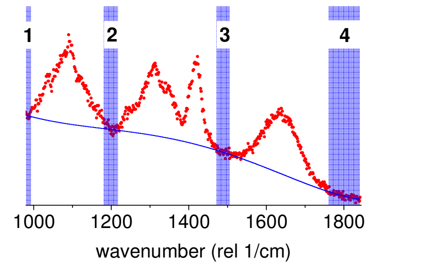	981 – 992	1178 – 1217	1475 – 1504	1763 – 1826

The peak fitting itself consisted of fitting the COO^- symmetric vibration of sodium alginate at position ~1415 rel. cm^{-1} and water bending vibration at 1640 rel. cm^{-1}. Both alginate and water vibrations were fitted with one Gauss function without the linear offset. The regions used for data fitting with these functions were 1386 – 1454 rel. cm^{-1} for alginate vibration and 1503 – 1800 rel. cm^{-1} for water bending vibration.

In the case of multicomponent microcapsule made of sodium alginate (SA), sodium cellulose sulfate (SCS), and poly(methylene-co-cyanoguanidine) (PMCG), the spectrum for fitting SA was processed in the same way as for alginate microbeads (**Table SN1.1**). The background subtraction for SCS and

PMCG bands was performed in the regions where the shape function was performed as shown in **Table SN1.2**:

Table SN1.2: Polynomial fitting for background subtraction in CRM imaging of the multi-component SA-SCS-PMCG microcapsule

<table>
<tr><td rowspan="3">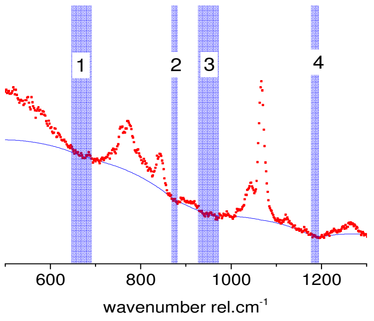
</td><td colspan="4">Regions for background subtraction
(rel. cm^{-1})</td></tr>
<tr><td>1</td><td>2</td><td>3</td><td>4</td></tr>
<tr><td>645 – 690</td><td>866 – 884</td><td>926 – 976</td><td>1176 – 1198</td></tr>
</table>

The vibration used for fitting the SCS peak corresponds to the symmetric stretching vibration of **v_s(O=S=O)** at the position of ~1070 rel. cm^{-1}.[1] The vibrations for PMCG have not been described so far in the literature. In this experiment the vibration at the position of 770 rel. cm^{-1} was used for fitting. Both SCS and PMCG vibrations were fitted with PseudoVoigt without the linear offset.

Supplementary Note 2: Calibration of Raman signal obtained from CRM imaging to alginate concentration in solution

The Raman signal from CRM imaging of alginate microbeads was transformed to absolute concentration of alginate chains *via* calibration of this signal to a known concentration of alginate in stock solutions. The SA concentration in the range of 1 to 6 wt.% (saline solutions) was used for calibration. In order to prepare the solutions of high SA concentration in the range > 3 wt.%, SA was degraded by sonication of the SA solution (see below). The CRM spectra were acquired from the solutions placed in a Petri dish. The signal was gathered by direct immersion of the objective into the solution. Between 64 and 128 sampling points were used with the exposure time between 2 and 10 s and up to three accumulations for each concentration. The details of these measurements are contained in **Table SN2.1**.

The areas under the curves were used to assess the quantitative relationship between the absolute concentration of alginate and measured signal. All spectra were normalized before further processing by dividing areas of alginate and water bands,[2] which provided the internal standardization for each alginate concentration and removed the effects related to laser energy and focal volume fluctuations. The medians of normalized peak areas for every concentration were fitted with a linear function for both the laser lines of 785 and 532 nm (**Fig. S7a** and **b**). Linear fits of percentiles (5% and 95%) for each concentration were employed as confidence bands for the error evaluation of SA concentration.

Sonication of sodium alginate. 150 mL of 1 wt.% SA solution in water was placed to a 250 mL beaker and fixed into the sonication setup (Bandelin Sonoplus HD 2070, Germany) and sonicated at room temperature for 40 min. Sonication power was applied as a sequence of 0.8 s pulses interrupted by 0.2 s

intervals using a sonication tip with nominal power of 500 W/cm^2. The sonicated SA solution was subsequently cooled to 4°C, precipitated in 1 L of 96% ethanol and placed in a refrigerator for 12 h. Ethanol was replaced three times at 8 h intervals. Precipitated SA was dried overnight at 40 °C, followed by grinding in a mortar and storage at room temperature. The water content was 8%.

Table SN2.1. **Conditions for collecting the Raman spectra of sodium alginate solutions in saline used for calibration of Raman signal to absolute concentration of alginate. *N* defines the number of sampling points.**

Laser line

785 nm

Concentration (wt.%)	Collection time (s)	Batch no.	*N* total	*N* total	*N* total
1	5	1	64	64	64
2	5	1	64	64	64
3	5	1	64	64	64
4	5	1	64	64	128
	5	2	64	64	
5	5	1	64	128	384
	5	1	64		
	5	2	64	128	
	5	2	64		
	5	3	64	128	
	5	3	64		
6	5	1	64	128	384
	5	1	64		
	5	2	64	128	
	5	2	64		
	5	3	64	128	
	5	3	64		

532 nm

Concentration (wt.%)	Collection time (s)	Batch no.	*N* total	Batch no.	*N* total	*N* total
1	2	1	36	1	108	215
	2	2	36			
	5	1	36			
	5	2	35	2	107	
	10	1	36			
	10	2	36			
2	2	1	36	1	108	252
	2	2	36			
	2	2	36			
	5	1	36			
	5	2	36	2	144	
	10	1	36			
	10	2	36			
3	2	1	36	1	108	216
	2	2	36			
	5	1	36			
	5	2	36	2	108	
	10	1	36			
	10	2	36			
4	2	1	36	1	108	108
	5	1	36			
	10	1	36			
5	5	2	36	2	72	72
	10	2	36			
6	2	2	36	2	72	72
	10	2	36			

Once the linear fitting was done, the inverse formulae to retrieve the relevant concentration values were applied (graphical representation for the laser line 532 nm is shown in **Fig. S7c**). The predicted upper and lower limits $\hat{x}_{95}$ and $\hat{x}_5$ were derived as follows:

$$\hat{x}_{95} = {(\delta + \varphi\hat{x} - \alpha)}/{\beta} \quad \hat{x} = {(y - \alpha)}/{\beta} \quad \hat{x}_5 = {(\gamma + \eta\hat{x} - \alpha)}/{\beta}$$

where $\hat{x}$ is prediction of concentration, δ and ϕ are intercept and slope, respectively, of linear fit at 95% percentile, γ and η are intercept and slope, respectively, of linear fit at 5% percentile, and α and β are intercept and slope, respectively, of linear fit of concentration. Consequently, all the values of the local alginate concentration are expressed as $\hat{x}$ values with $\hat{x}_{95}$ and $\hat{x}_5$ values, expressing the error bar of the determined absolute concentrations. The limit of detection (LOD) was estimated to be 0.29 wt.% at 785 nm laser excitation line. It was calculated as an intersection of δ and $\hat{y}_5$ in the calibration curve. Type I and II error rates were set to 5%. The evaluation of LOD was adapted from Lavagnini and Magno.[3] Additionally, the limit of quantitation of 1.14 wt.% at 785 nm was evaluated according Armbruster et al.[4] setting the coefficient of variation equal to 17%.

REFERENCES

1. Zhang, K., Brendler, E., Geissler, A., Fischer, S. Synthesis and spectroscopic analysis of cellulose sulfates with regulable total degrees of substitution and sulfation patterns via 13C NMR and FT Raman spectroscopy. *Polymer*, 52, 26-32 (2008).
2. Heinemann, M., Meinberg, H., Büchs, J., Koß, H.-J., Ansorge-Schumacher, M. B. Method for quantitative determination of spatial polymer distribution in alginate beads using Raman spectroscopy. *Appl. Spectrosc.*, 59, 280-285 (2005).
3. Lavagnini, I., Magno, F. A statistical overview on univariate calibration, inverse regression, and detection limits: Application to gas chromatography/mass spectrometry technique. *Mass Spectrom. Rev.*, 26, 1-18 (2007).
4. Armbruster, D.A., Pry, T. Limit of Blank, Limit of Detection and Limit of Quantitation. *Clin. Biochem. Rev*. 29 (Suppl 1), S49-S52 (2008).
